# Phylodynamics reveals the role of human travel and contact tracing in controlling the first wave of COVID-19 in four island nations

**DOI:** 10.1093/ve/veab052

**Published:** 2021-06-08

**Authors:** Jordan Douglas, Fábio K Mendes, Remco Bouckaert, Dong Xie, Cinthy L Jiménez-Silva, Christiaan Swanepoel, Joep de Ligt, Xiaoyun Ren, Matt Storey, James Hadfield, Colin R Simpson, Jemma L Geoghegan, Alexei J Drummond, David Welch

**Affiliations:** Centre for Computational Evolution, The University of Auckland, Auckland 1010, New Zealand; School of Computer Science, The University of Auckland, Auckland 1010, New Zealand; Centre for Computational Evolution, The University of Auckland, Auckland 1010, New Zealand; School of Biological Sciences, The University of Auckland, Auckland 1010, New Zealand; Centre for Computational Evolution, The University of Auckland, Auckland 1010, New Zealand; School of Computer Science, The University of Auckland, Auckland 1010, New Zealand; Centre for Computational Evolution, The University of Auckland, Auckland 1010, New Zealand; School of Computer Science, The University of Auckland, Auckland 1010, New Zealand; Centre for Computational Evolution, The University of Auckland, Auckland 1010, New Zealand; School of Biological Sciences, The University of Auckland, Auckland 1010, New Zealand; Centre for Computational Evolution, The University of Auckland, Auckland 1010, New Zealand; School of Computer Science, The University of Auckland, Auckland 1010, New Zealand; Institute of Environmental Science and Research Limited (ESR), Poriua 5420, New Zealand; Institute of Environmental Science and Research Limited (ESR), Poriua 5420, New Zealand; Institute of Environmental Science and Research Limited (ESR), Poriua 5420, New Zealand; Vaccine and Infectious Disease Division, Fred Hutchinson Cancer Research Center, Seattle, Washington WA 98109-1024, USA; School of Health, Victoria University of Wellington, Wellington 6012, New Zealand; Institute of Environmental Science and Research Limited (ESR), Poriua 5420, New Zealand; Department of Microbiology and Immunology, University of Otago, Dunedin 9016, New Zealand; Centre for Computational Evolution, The University of Auckland, Auckland 1010, New Zealand; School of Computer Science, The University of Auckland, Auckland 1010, New Zealand; School of Biological Sciences, The University of Auckland, Auckland 1010, New Zealand; Centre for Computational Evolution, The University of Auckland, Auckland 1010, New Zealand; School of Computer Science, The University of Auckland, Auckland 1010, New Zealand

**Keywords:** COVID-19, coronavirus, phylogenomics, human movement, contact tracing, New Zealand, Australia, Taiwan, Iceland

## Abstract

New Zealand, Australia, Iceland, and Taiwan all saw success in controlling their first waves of Coronavirus Disease 2019 (COVID-19). As islands, they make excellent case studies for exploring the effects of international travel and human movement on the spread of COVID-19. We employed a range of robust phylodynamic methods and genome subsampling strategies to infer the epidemiological history of Severe acute respiratory syndrome coronavirus 2 in these four countries. We compared these results to transmission clusters identified by the New Zealand Ministry of Health by contact tracing strategies. We estimated the effective reproduction number of COVID-19 as 1–1.4 during early stages of the pandemic and show that it declined below 1 as human movement was restricted. We also showed that this disease was introduced many times into each country and that introductions slowed down markedly following the reduction of international travel in mid-March 2020. Finally, we confirmed that New Zealand transmission clusters identified via standard health surveillance strategies largely agree with those defined by genomic data. We have demonstrated how the use of genomic data and computational biology methods can assist health officials in characterising the epidemiology of viral epidemics and for contact tracing.

## 1. Introduction

Many continued hoping that the epidemic would soon die out and they and their families be spared. Thus they felt under no obligation to make any change in their habits, as yet. Plague was an unwelcome visitant, bound to take its leave one day as unexpectedly as it had come.

Albert Camus (‘The Plague’, 1947)

The respiratory tract illness caused by Severe acute respiratory syndrome coronavirus 2 (SARS-CoV-2) known as Coronavirus Disease 2019 (COVID-19; (Rothan and Byrareddy 2020)) has caused substantial global health and economic impact, and the global epidemic appears far from over. COVID-19 was first detected in the city of Wuhan, Hubei province (China) in late 2019 (Zhu et al. 2020). By 11 March 2020 there were 100,000 cases across 114 countries leading the World Health Organization (WHO) to declare a pandemic (World Health Organization 2020a). By early 2021, over 2 million deaths had been reported and over half of the world’s population had received stay-at-home orders (Alasdair Sandford 2020).

COVID-19 has now been characterised extensively. The average number of people each case goes on to infect (known as the effective reproduction number, *R*_e_), for example, has been estimated as 1–6 during the early stages of the outbreak (Liu et al. 2020; Binny et al. 2020a; Alimohamadi, Taghdir, and Sepandi 2020). Because COVID-19 often causes mild symptoms Day (2020a,b) and surveillance efforts are variable, the difference between the true and reported number of cases could be of an order of magnitude or more (Ruiyun et al. 2020a; Lu et al. 2020). Fortunately, many locations have demonstrated that it is possible to reduce *R_e_* to less than 1 as a result of mitigation efforts including contact tracing, case isolation, and travel restrictions (Binny et al. 2020a; Chinazzi et al.).

As we reflect on the early months of the pandemic (until the end of April 2020), different parts of the world tell contrasting stories about their struggle against COVID-19 (Table 1).

**Table 1.** Summary of the COVID-19 pandemic in the four target islands. Total passenger arrivals into the island in the January–March period are counted. All dates are in the Year 2020. The number of confirmed cases and the percentage of cases with recent overseas travel are reported for the January–April period.

Island	Population (millions)	Arrival (millions)	First case	Border close	Confirmed cases	Overseas travel
New Zealand	5	1.6 (Stats NZ 2020)	February 28	March 19 (RNZ 2020)	1,129 (World Health Organization 2020b)	39% (New Zealand Ministry of Health, Manatu Hauora 2020)
Australia	25	4.3 (Australian Bureau of Statistics, 2020)	January 25	March 20 (Burke 2020)	6,746 (World Health Organization 2020b)	64% (Department of Health 2020)
Iceland	0.34	0.33^a^ (Icelandic Tourist Board 2020)	February 28	March 20 (Schengen Visa Info 2020)	1,797 (World Health Organization 2020b)	19% (The Directorate of Health, The Department of Civil Protection, and Iceland 2020)
Taiwan	23	2.6 (Strong 2020)	January 21	March 19 (Aspinwall 2020)	429 (World Health Organization 2020b)	90% (Taiwan Centers for Disease Control 2020a)

a Visitor arrivals only.

Aotearoa New Zealand, Australia, Iceland, and Taiwan, for example, fared comparatively well against their ‘first wave’, with smaller infection peaks and lower excess mortalities compared to many mainland countries (World Health Organization 2020b; FT Visual 2020), with New Zealand and Taiwan achieving elimination or near-elimination of their first waves in that period (Cousins, 2020; Summers et al. 2020). Over the following year, all four locations experienced ‘second waves’ of community transmission (Aljazeera 2020a, 2020b; Iceland Review 2020; The Guardian 2021).

Irrespective of their current COVID-19 situation, these four countries share in common the effectiveness of their response to the first outbreak, which contributed positively to their swift recovery at the time. In March 2020 amidst the first wave, all four nations closed their borders and required isolation or quarantine of those who were allowed through (Hale et al. 2020), either restricting or discouraging human movement (Apple 2020). Stay-at-home orders were issued in New Zealand and Australia but not in Iceland or Taiwan (Hale et al. 2020). Stringent contact tracing, coupled with isolation and/or quarantine of cases and their close contacts, proved to be effective strategies in these four locations (Baker et al. 2020; Draper et al. 2020; Gudbjartsson et al. 2020a; Jason Wang, Ng, and Brook 2020). These islands thus provide excellent case studies to characterise the effects of human travel on epidemic spread during the early months of the pandemic, a critical endeavour when facing the threat of global endemicity.

**Figure 1.**
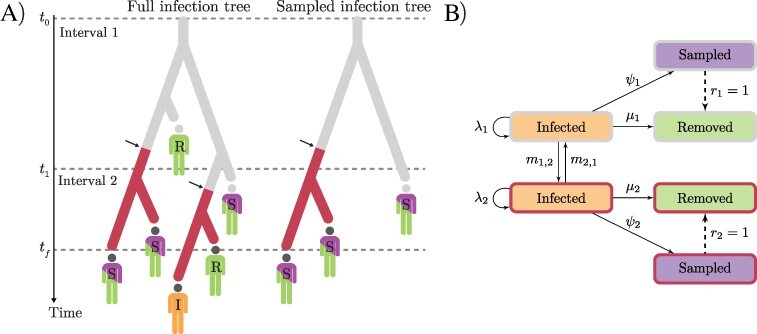
Depiction of the MTBD model. (A) A full infection tree and a sampled infection tree (*T*), with two time intervals (epochs) and two demes (indicated in grey and red). All lineages in the trees represent infected individuals (*I*). Branching corresponds to transmission and colour change (arrows) corresponds to migration; infected individuals may be removed (*R*) or sampled and then removed (*S*). (B) Transition diagram: solid and dashed arrows labelled with rates and probabilities, respectively.

Mathematical methods for studying epidemics are as diverse as the diseases they target, making varied use of surveillance and genetic data and ranging from models of mathematical epidemiology (Grassly and Fraser 2008; Binny et al. 2020a; Ferretti et al. 2020; Hethcote 2000) to phylodynamic models (Volz, Koelle, and Bedford 2013). The latter take advantage of the comparable timescales of epidemiological and evolutionary processes (Grenfell et al. 2004), which result from short viral generation times and high viral mutation rates (20–40 mutations per year for SARS-CoV-2; (Lai et al. 2020; Xingguang et al. 2020b; Rambaut 2020)). By modelling the transmission process as phylogenetic trees (Fig. 1), phylodynamic and molecular evolution models can be coupled to reconstruct the mutational history of viral genomes (Xingguang et al. 2020b; Rambaut 2020; Lai et al. 2020), thereby elucidating the epidemiological processes that shaped them.

In this study, we considered epidemic dynamics in New Zealand, Australia, Iceland, and Taiwan over the first few months of the pandemic up until the end of April. The goal was to determine how SARS-CoV-2 case numbers varied as human mobility changed in response to government restrictions. We incorporated human movement into our analyses through the modelling of mobile phone data. More specifically, we hypothesised that introductions of SARS-CoV-2 would sharply decline following international travel and that viral transmission (*R_e_*) would decline following human movement.

While the notion that restricting human movement constrains the spread of infectious diseases may appear exhaustively addressed, our study is unique in its integration of human mobility data with genomic and epidemiological data, in geographically isolated island settings. There is a great benefit in studying the spread of disease in these locations, as their geographic isolation reduces the effect of unregulated and undocumented land border crossing that is common to mainland countries. We do so by analysing viral genomic data under a range of phylogenetic methods, while at the same time controlling for data subsampling strategies. Lastly, we compare the phylodynamic history of New Zealand’s first outbreak with that inferred by the New Zealand Ministry of Health (NZMH) in order to assess the ability of contact tracing at identifying transmission pathways.

## 2. Methods

In the studyof disease phylodynamics, a complete epidemic can be modelled as a rooted, binary phylogenetic tree whose *N* tips are all infected individuals (Fig. 1) and whose characteristics and drivers we wish to infer. In a rapidly growing epidemic, the age of this tree serves as a good proxy for the duration of the epidemic. Because it is seldom possible to sample the entire infected population, one instead reconstructs a subtree *T* from a sample of cases of size *n*, where *n < N*. This sample provides *n* viral genome sequences we refer to as *D*, as well as each sequence’s sampling date, collectively represented by vector *y. D* and *y* thus constitute the input data and are herein referred to as ‘samples’. In the case of structured phylodynamic models, samples in *y* are further annotated by ‘deme’—a geographical unit such as a city, a country, or a continent.

A Bayesian phylodynamic model is characterised by the following unnormalised posterior probability density function:

(1) \begin{equation*}f({\rm {T}},\mu_{c},\theta_{s},\theta _{\tau}|D,y) \propto {\rm{P}}(D|{\rm {T}},{\mu _c},{\theta _s})\;f({\rm {T}}|{\theta _\tau })\;{f_{pr}}({\mu _c},{\theta _s},{\theta _\tau }),\end{equation*}

where P(*D*|T*,µ_c_,θ_s_*) is the phylogenetic likelihood of tree *T* given alignment *D*, whose sequences accumulate substitutions at rate *µ_c_* under a substitution model with parameters *θ_s_. f*(T|*θ_τ_*) is the probability density function of the tree prior sampling distribution, given tree prior parameters *θ_τ_* (in this study we consider a range of phylodynamic models that differ in their tree prior; see sections 2.1 and 2.2 and Supplementary materials). Finally, *f_pr_*(*µ_c_,θ_s_,θ_τ_*) is the prior distribution on the remaining parameters.

### 2.1 Multitype birth–death model

The multitype birth–death (MTBD) model (Kuhnert et al. 2016; Sciré et al. 2020) is a structured phylodynamic model that assumes infected (‘I’; Fig. 1) hosts of viral lineages are organised into a set of demes (‘types’) and migrate between them at rate *m* (Fig. 1). Infected individuals transmit the infection at birth rate *λ* and can be sampled (‘S’; Fig. 1) and sequenced at sampling rate *ψ*; after which they are removed (‘R’; Fig. 1) with probability *r*. We set *r *= 1 by assuming (sampled) confirmed cases are isolated and are unlikely to cause further infections. Removal without sampling happens at death rate *µ* (Fig. 1). Here, ‘death’ refers to anything causing hosts to no longer be infectious, including recovery with immunity, host death, or behavioural changes (e.g. self-isolation).

The probability density function of *T* under the MTBD sampling distribution (Kuhnert et al. 2016) and its parameters *θ_τ_* is given by:

(2) \begin{equation*}f({\rm {T}}|{\theta _\tau }) = f({\rm {T}}|\lambda ,\mu ,\psi ,m,r,{\pi _G},t,{\mathcal O}),\end{equation*}

where *π_G_* represents the frequency of lineages in each deme at the root and *O* is tree *T*’s age.

The MTBD model is reparameterised into a computationally and epidemiologically convenient form, with parameters ${R_e} = {\lambda \over {\mu + \psi r}},b = \mu + \psi r$, and $S = {\psi \over {\psi + \mu }}.\;{R_e}$ is the disease’s effective reproductive number (Stadler et al. 2013). *b* is the total rate at which individuals become non-infectious, and *s* is the probability of an individual being a sample (out of all removed individuals). The phylodynamic tree prior under this parameterisation is

(3) \begin{equation*}f({\rm {T}}|{\theta _\tau }) = f({\rm {T}}|{R_{\rm{e}}},b,s,m,r,{\pi _G},t,{\mathcal O}).\end{equation*}

### 2.2 Alternate phylogenetic models

As a sensitivity analysis, we invoke three additional phylogenetic models on top of MTBD and compare the results between the four models. These models are (1) a discrete phylogeography model (DPG, Lemey et al. (2009)), (2) a discrete phylogeography model with informed effective population sizes and epochs (DPG+), and (3) the structured coalescent model with informed effective population sizes and epochs (SC, Muller, Rasmussen, and Stadler (2018)). Details of these models are in Supplementary information.

### 2.3 Demes and infection time intervals

We consider four independent geographical models, each characterised by two demes: a target island deme (New Zealand, Australia, Iceland, or Taiwan; referred to as the ‘IS’ deme) and a mutually exclusive ‘rest-of-the-world’ deme (‘RW’) that contributes samples from other countries in the world. All RW-specific parameters are considered nuisance parameters.

Under our MTBD model, *R_e_, b, s*, and *m* are estimated across a series of time intervals (and held constant within intervals) defined by time boundaries *t*, with *t*_0_ *< t*_1_ *< … < t_f_*, where *t* is fixed at informed dates. *t*_0_ is the beginning of the infection, and *t_f_* is both the last sampling time and the end of the last interval (Fig. 1). Time intervals are both parameter- and deme-specific.

*R_e_* and *b* are modelled with three epochs *t*_0_ *< t*_1_ *< t*_2_ *< t_f_*, where *t*_1_ is the first reported case in each deme and *t*_2_ is the point of human mobility decline, according to a Bayesian hierarchical sigmoidal model trained on Apple mobility data (see Results and Supplementary information). These mobility data consist of human driving, walking, and public transit use as tracked through Apple mobile phone user movement. Epoch dates for *s* and *m* can be found in Table S3.

### 2.4 SARS-CoV-2 genomic data and subsampling

We sequenced and assembled 217 New Zealand SARS-CoV-2 genomes (representing 19 per cent of confirmed cases during the relevant time period) that are now available on Global Initiative on Sharing All Influenza Data (GISAID; Shu and John 2017), which provided the remaining 3,000+ genome sequences analysed here. Due to computational limitations, we subsampled GISAID sequences as follows: (1) two different subsample sizes (‘small’ vs. ‘large’), with 200 and 500 RW (‘rest of the world’ deme) sequences, and (2) two sample selection methods (‘time’ vs. ‘active’) that chose sequences uniformly across time or proportional to the number of active cases over time. Up to 250 sequences from the IS (island) deme are included in each alignment (where more than 250 were available, we subsampled them using the respective sampling method). We restricted our analyses to the early months of the pandemic (until the end of April 2020) because this time window encompasses largely resolved (i.e. from onset to end) outbreaks in all four countries, while also making data set sizes more manageable and precluding further subsampling and thinning of data points.

Permuting the above two options yields four subsampling protocols, which were applied to the four IS demes, generating a total of sixteen SARS-CoV-2 alignments (30-kb long, 0.3 per cent ambiguous sites on average). Each alignment consists of sequences from one of the four islands (IS) and from the rest of the world (RW). Moreover, each alignment was analysed with each phylodynamic model (MTBD, SC, DPG, and DPG+), coming to a total of forty-eight Bayesian phylogenetic analyses.

## 3. Results

### 3.1 Modelling human movement as tracked by mobile phone data

**Figure 2.**
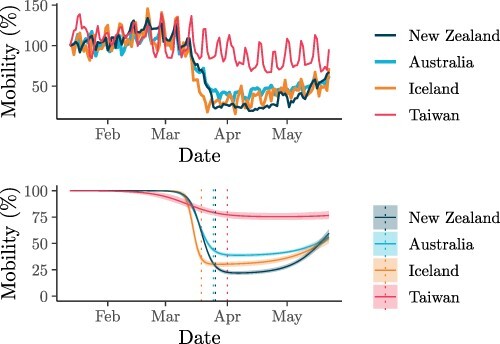
Human walking movement relative to Day 1 (100 per cent). Data were obtained by tracking the mobility of Apple mobile phone users (Apple 2020). Top: movement data from Apple. Bottom: fitted sigmoid model (solid lines show the median and shaded curves show the 2.5–97.5 per cent quantile interval); vertical dotted lines correspond to the estimated mean date of mobility change, which defines the *t*_2_ epoch date for each deme.

With the goal of characterising the decrease in human movement over the weeks our SARS-CoV-2 samples were collected; we employed a novel Bayesian hierarchical sigmoidal model for the analysis of mobile phone data (Apple 2020; Fig. 2). This analysis revealed that human movement generally decreased in all four islands, with Taiwan being the first to show a declining trend among the islands, but also the least pronounced one. New Zealand, Australia, and Iceland underwent a marked decline in human movement during March 2020, with a slight recovery starting around mid-April. Weekly travel oscillations can be observed in the original mobility data, but are averaged out by the sigmoidal model.

The dates of mobility reduction provided by this mobility model informed the start of the final time interval *t*_2_ in our three-epoch models of *R_e_* and *b*.

### 3.2 SARS-CoV-2 phylodynamics

We tested four genome subsampling regimes under four phylodynamic models. This sensitivity analysis suggests that inference under the MTBD and SC models is fairly robust to subsampling protocols, as opposed to DPG and DPG+, which yielded unrealistically small estimates of infection duration and of viral introduction counts (see Iceland; Fig. 5), particularly from the ‘active’ method. Such model behaviour is unsurprising and has been reported elsewhere (Nicola et al. 2015), and it was only slightly alleviated when ‘large’ subsamples were used.

Analyses under MTBD and SC were prohibitively time-demanding using the larger data sets. For consistency, we only present results from the ‘small-time’ data set in the main article, but further results can be found in Supplementary information. Four ‘small-time’ data sets consist of up to 250 sequences from the target deme IS and 200 sequences sampled uniformly through time from the rest-of-the-world deme RW.

**Figure 3.**
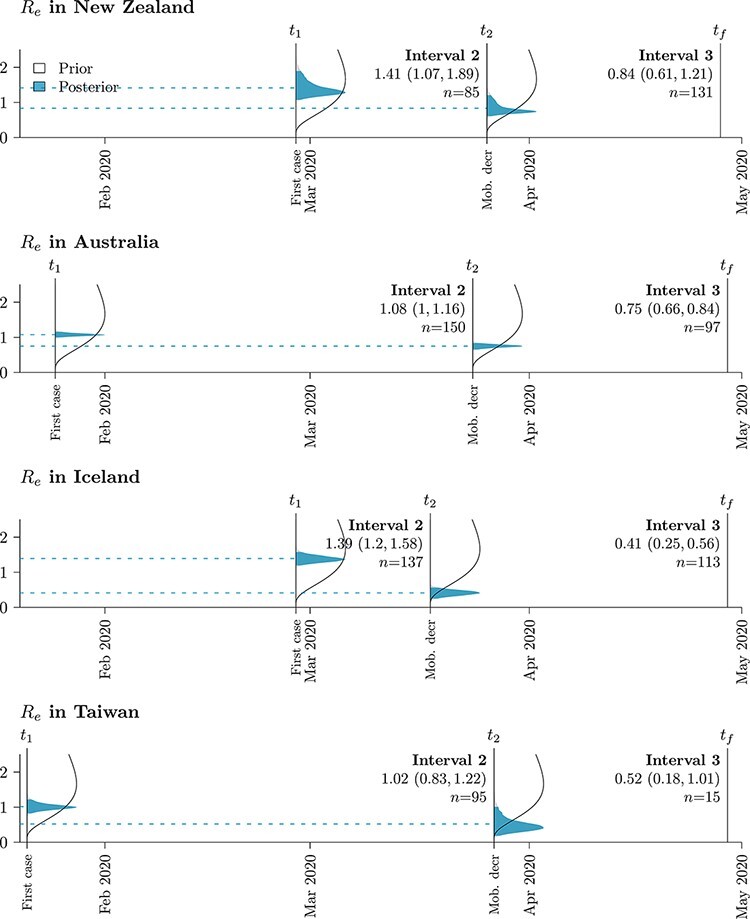
*R_e_* prior (white) and posterior (blue) probability distributions across islands and time intervals (*n* samples in each interval). Posterior means are indicated by dashed lines. Mean estimates (and 95 per cent highest posterior density intervals) are indicated under each time interval: *t*_1_ is the first case in the target deme, *t*_2_ the start of mobility reduction, and *t_f_* the final sample in the data set.

**Figure 4.**
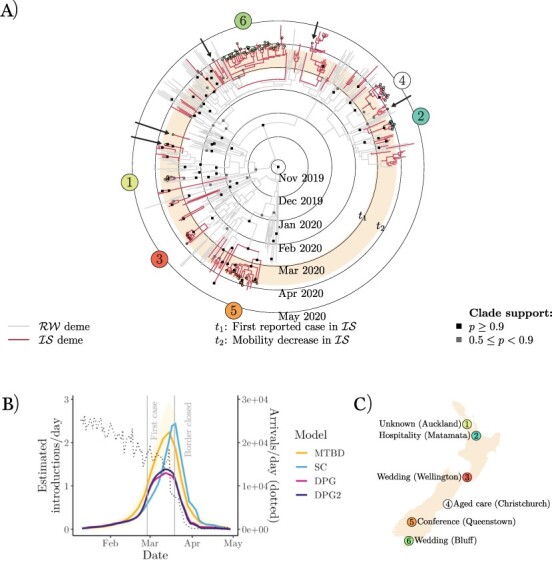
(A) New Zealand COVID-19 maximum-clade-credibility tree (from ‘small-time’ alignment). Samples are labelled according to outbreaks identified by NZMH. Arrows indicate cases assigned to a cluster by NZMH, which are not monophyletic with the rest of the cluster. (B) Number of SARS-CoV-2 introductions in New Zealand over time. (C) New Zealand cluster locations (with greater than five cases).

**Figure 5.**
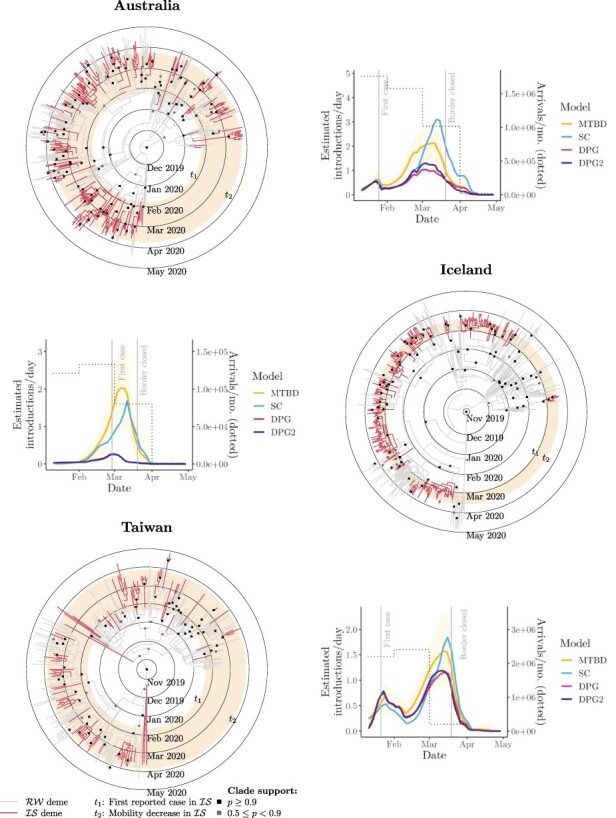
Maximum-clade-credibility COVID-19 tree (from ‘small-time’ alignment) for Australia (top), Iceland (middle), and Taiwan (bottom). Plots show the estimated number of SARS-CoV-2 introductions over time.

We estimated *R_e_* independently for each epoch using the MTBD model (Fig. 3). This analysis estimates *R_e_* as 1.02–1.41 during the early infection of each respective country (following the first case) and later at less than 1 (following human mobility reduction). These estimates were not sensitive to sampling strategies. Each island IS saw multiple introductions of SARS-CoV-2 from the rest of the world (RW; Figs. 4 and 5). Under MTBD, we estimated the mean number of sample sequence introductions to be 63, 87, 49, and 65 for New Zealand, Australia, Iceland, and Taiwan, respectively. Conversely, we estimated just one virus export event from each country to the rest of the world, across the time period and sample considered. Similar results were observed using the SC model, with neither SC nor MTBD producing drastically different estimates between subsampling strategies.

The larger estimates of *R_e_* for New Zealand and Iceland (∼1.4 before mobility decline) reflect their samples having larger proportions of community transmission, as opposed to viral introduction events, in comparison to Australia and Taiwan, which have smaller *R_e_* estimates (∼1). The genome samples themselves are a product of the public health surveillance policy within each country, and non-random sampling of cases could lead to biassed estimates of *R_e_*; for example, intensive sampling of large outbreaks would inflate *R_e_* and increased sequencing of overseas returnees is likely to deflate *R_e_*. All countries studied here practised widespread sampling and sequencing of cases, which would produce relatively unbiased samples.

### 3.3 Comparison with contact tracing in New Zealand

The NZMH defines a significant COVID-19 cluster to comprise ten or more cases connected through transmission and who are not all part of the same household (New Zealand Ministry of Health 2020). Five of these clusters are represented by five or more of our samples (Fig. 4A and C), with a sixth non-significant cluster (based in the city of Auckland).

A clade is defined as a set of samples (within a phylogenetic tree) that share an exclusive common ancestor. Here we define a phylogenetic cluster as a clade whose samples share a cluster label and whose origin can be explained by invoking a single introduction into a focal deme. Under this definition (1) case exports are allowed, (2) isolated labelled tips outside of the clade are considered type-1 contact tracing mismatches (i.e. contact tracers assigned a case to a cluster they were not genomically linked to), and (3) unlabelled tips that are both from the focal deme and placed within the clade are considered type-2 mismatches (i.e. genomics assigned a case to a cluster when contact tracers could not).

Using this definition, all clusters identified by NZMH were recovered (among those represented by our samples). However, there were five type-1 mismatches: two from an Auckland-based cluster, one from Christchurch, one from Queenstown, and one from Bluff (shown by arrows in Fig. 4). We estimatedeighteen type-2 mismatches. Our alternative subsampling schemes produced similar results: estimating 5, 4, and 7 type-1 mismatches and 18, 19, and 18 type-2 mismatches for ‘small-active’, ‘large-time’, and ‘large-active’, respectively. This suggests that the method is fairly robust to subsampling strategy.

## 4. Discussion

In this article, we explored the introduction and transmission history of COVID-19 in New Zealand, Australia, Iceland, and Taiwan—geographically isolated countries that so far have been successful in preventing this disease from wreaking havoc as extensively as it did in other parts of the world. We also delved deeper into the outcome of contact tracing within New Zealand specifically. This study is novel in its integration of genomic, epidemiological, and mobile phone mobility data through explicit statistical models. The methods employed are robust and transparent in that they (1) explicitly justify and incorporate prior knowledge about epidemiological parameters (see Supplementary information), (2) report uncertainty in parameter estimation, and (3) explore the effect that different data subsampling protocols have on parameter estimation.

### 4.1 Phylodynamic modelling complements contact tracing

Identifying cases and the sources of their infections is an essential exercise in slowing infectious diseases such as COVID-19 (Hellewell et al. 2020), as recognised by the Director-General of the WHO in his pronouncement: ‘You cannot fight a fire blindfolded. And we cannot stop this pandemic if we don’t know who is infected’. Modern health surveillance strategies include medical databases containing travel history (Jason Wang, Ng, and Brook 2020), the development of mobile phone applications (Ferretti et al. 2020), and the identification of outbreak subgroups known as ‘clusters’, whose cases share the same origin (New Zealand Ministry of Health 2020). At the core of health surveillance practice lays contact tracing—the goal of which is to pinpoint the origin of infections by reconstructing the travel history of confirmed cases and identifying all individuals with whom cases have come in contact.

Nonetheless, contact tracing has its limitations. Establishing the link between cases is restricted, for example, by the ability of cases to recall their recent contacts and travel history, which can lead to type-2 mismatches between epidemiological and genetic clusters. Furthermore, a case may have attended an event causally linked to a cluster but acquired their infection elsewhere and did not infect anyone at the event (type-1 mismatches). The implications of these types of error are manifold. If cluster sizes are underestimated, then so too is the rate of disease spread. If the extent of import-related cases is overestimated, then the impact of international air travel and the extent of community transmission cannot be fully accounted for.

After considering 217 genomes (representing 19 per cent of the cases in New Zealand at the time), our results suggest that there are additional eighteen unclassified cases in New Zealand that could have been linked to known clusters but were not (type-2 mismatch) and five cases that were linked to a cluster where they did not acquire or transmit the infection (type-1). However, by and large, our phylodynamic analysis is in agreement with conclusions reached by NZMH.

Overall, we have shown that contact tracing has been accurate among the cases considered and succeeded to a large degree in identifying individuals belonging to the same infection cluster. We have demonstrated how the rapid real-time availability and assessment of viral genomic data can complement and augment health surveillance strategies and thus improve the accuracy of contact tracing methods.

As the pandemic has progressed, real-time whole-genome sequence data have been integrated into the health response of the four countries discussed here (Geoghegan et al. 2021; Seemann et al. 2020; Gudbjartsson et al. 2020b; Taiwan Centers for Disease Control 2020b).

Genome sequencing is increasingly affordable and shared global surveillance resources such as NextStrain (Hadfield, et al. 2018) and GISAID are becoming more complete and useful. We advocate widely incorporating modern methods of genetic epidemiology into standard contact tracing and epidemic surveillance in order to ensure that COVID-19 and future epidemics leave as promptly as they come.

### 4.2 Restricting human movement constrains COVID-19 spread

As we have demonstrated for all four countries, genomic data can be used in the estimation of disease introductions over time, a goal particularly hard to achieve with contact tracing alone. Our results suggest that SARS-CoV-2 was introduced into each island on many independent occasions, corroborating the available epidemiological data from health officials (Table 1). Viral imports rapidly declined around the time of border closure in mid-late March 2020 as a result of decreased international travel (Figs. 4 and 5). We believe this in turn played a significant role in limiting the spread of COVID-19.

In contrast, even with plentiful genomic data, it is non-trivial to reliably estimate the number of exportation events. Such an estimate would depend on a large, unbiased global sample that is challenging to obtain and whose analysis is prohibitively slow.

Mobile phone data revealed idiosyncratic human movement patterns (Fig. 2). During the early stages of the pandemic, human mobility rapidly declined in both New Zealand and Australia as a result of stay-at-home orders (Hale et al. 2020), as well as in Iceland despite no such official restraints. Taiwan, on the other hand, did not exhibit as much of a decline in human movement nor did it enforce stay-at-home orders on the general population. A goal of this study was to characterise the extent to which this decline in human movement could impact the spread of COVID-19.

We estimated effective reproduction numbers, *R_e_*, as approximately 1–1.4 at the onset of the pandemic in all four islands (Fig. 3). These estimates are not as high as in other countries where the pandemic was more widespread and where *R_e_* was typically estimated in the range of 2–6 (Liu et al. 2020; Binny et al. 2020a; Alimohamadi, Taghdir, and Sepandi 2020). Our low estimates reflect the fact that a large fraction of cases in these countries were travel-related imports and that community transmission rates were low. *R_e_* declined as human movement decreased in each of the countries studied, reaching values below 1.0 (Fig. 3). Estimates of *R_e_* based only on epidemiological data are also consistently below 1 during these time periods (Binny et al. 2020a,b; Price et al. 2020; Karnakov et al. 2020). For New Zealand, Australia, and Iceland, the decline in *R_e_* by 30–70 per cent coincided with a decline in human mobility of 50–80 per cent.

Our findings suggest that the first wave of COVID-19 in the four islands was controlled by greatly reducing international travel coupled with strong reductions in domestic movement. While the human movement data tell us nothing about the role contact tracing played in the four islands, it is noted that traditional contact tracing methods are insufficient in themselves (Ferretti et al. 2020) and that contact tracing systems were not well established across all territories early in the pandemic (Verrall 2020). The exception to this may be Taiwan, where an extreme restriction of domestic human movement was not required for suppressing a pandemic like COVID-19. In the first three months, Taiwan saw fewer cases-per-capita than New Zealand by an order of magnitude, for instance (Table 1), despite the latter enforcing a strict nationwide lockdown while having lower population density. Taiwan’s success has been attributed to the Taiwan Centers for Disease Control (Taiwan CDC) who were well prepared for such an epidemic following their experience with the SARS-CoV-1 outbreak in 2003—the early implementation of mass masking in public spaces and border control (Summers et al. 2020; Jason Wang, Ng, and Brook 2020).

Nevertheless, a stringent mobility-reduction response against COVID-19, likely contributed to SARS-CoV-2 elimination in New Zealand by May 9 (Cousins, 2020), thus being the first country with over 1,000 confirmed cases to return to zero active cases, allowing it to re-open its economy, albeit with a restricted border. Most importantly, human morbidity and mortality were limited. Conversely, while Australia also saw a decline in human mobility and initially enjoyed some measure of success against COVID-19, its more lenient approach, compared with either New Zealand’s or Taiwan’s, might underlie Australia’s longer path to elimination (Hale et al. 2020; Aljazeera 2020b).

In conclusion, we have shown that the introduction of SARS-CoV-2 was significantly inhibited as a result of international travel reduction in March 2020—notably the closing of borders in the four islands examined. We have also shown that the spread of SARS-CoV-2 within these communities, while initially lower than other parts of the world, saw a further reduction following the decline of human mobility within the countries. However, positive outcomes against this and similar pandemics will be dependent on a multitude of other factors including contact tracing, case isolation, and hygienic practices (MacIntyre 2020). As such, none of these should be seen as a silver bullet.

## Supplementary Material

veab052_Supp

## Data Availability

For reproducibility, BEAST2 input files for the four phylogenomic models (with sequences removed) are available at https://github.com/CompEvol/Covid4Island.
