# Supplementary material for: Phylodynamics reveals the role of human travel and contact tracing in controlling the first wave of COVID-19 in four island nations
Source: Virus Evol. 2021 Jun 8;7(2):veab052. doi: 10.1093/ve/veab052 (PMC8344840; doi:10.1093/ve/veab052)
Supplement: veab052_Supp [file veab052_supp.zip › supp_mat1.pdf]

Phylodynamics reveals the role of human travel and contact tracing in controlling the first wave of COVID-19 in four island nations

Jordan Douglas[1,2,†,*], Fábio K. Mendes[1,3,†,*], Remco Bouckaert[1,2], Dong Xie[1,2], Cinthy L. Jiménez-Silva[1,3], Christiaan Swanepoel[1,2], Joep de Ligt[4], Xiaoyun Ren[4], Matt Storey[4], James Hadfield[5], Colin R. Simpson[6], Jemma L. Geoghegan[4,7], Alexei J. Drummond[1,2,3], and David Welch[1,2]

[1]Centre for Computational Evolution, The University of Auckland, Auckland, New Zealand
[2]School of Computer Science, The University of Auckland, Auckland, New Zealand
[3]School of Biological Sciences, The University of Auckland, Auckland, New Zealand
[4]Institute of Environmental Science and Research, Wellington, New Zealand.
[5]Vaccine and Infectious Disease Division, Fred Hutchinson Cancer Research Center, Seattle, WA, USA
[6]School of Health, Victoria University of Wellington, Wellington, New Zealand
[7]Department of Microbiology and Immunology, University of Otago, Dunedin, New Zealand

[†]To whom correspondence should be addressed; Email: jordan.douglas@auckland.ac.nz, f.mendes@auckland.ac.nz.
[*]Authors contributed equally to this work.

SUPPORTING INFORMATION

Contents

1 **Viral genomic sequencing** 5

2 **Sequence preprocessing** 6

2.1 Filtering . . . 6

2.2 Subsampling . . . 6

2.3 Alignment . . . 7

3 **Demes and epochs** 8

3.1 A model of human movement decrease for mobile phone data . . . 9

4 **Model definition** 13

4.1 Substitution and clock models . . . 13

4.1.1 Partition schemes and substitution model selection . . . 13

4.2 Phylodynamic models . . . 13

4.2.1 Discrete phylogeography (DPG) . . . 13

4.2.2 Two epoch discrete phylogeography (DPG2) . . . 14

4.2.3 Structured coalescent (SC) . . . 15

4.2.4 Multi-type birth-death (MTBD) . . . 15

4.3 Prior distributions . . . 16

4.3.1 Prior for origin time . . . 16

4.3.2 Priors for sampling proportion s . . . 17

4.3.3 Priors for MTBD geographical frequencies π_G . . . 18

5 **Model implementation and parameter inference** 21

6 **Supplementary results** 25

6.1 Parameter estimates . . . 25

6.2 Introductions through time . . . 25

List of Figures

S1 Cell phone mobility data . . . 10
S2 Full MTBD probabilistic graphical model . . . 15
S3 Graphical summary of prior distributions . . . 20
S4 Clade posterior convergence . . . 24
S5 Posterior distribution of b over time . . . 26
S6 Posterior distribution of s over time . . . 27
S7 Posterior distribution of λ over time . . . 28
S8 Posterior distribution of ψ over time . . . 29
S9 Posterior distribution of μ over time . . . 30
S10 Comparison of mean root height and clock rate estimates . . . 31
S11 Comparison of the four subsampling methods for New Zealand alignments . . . 32
S12 Comparison of the four subsampling methods for Australia alignments . . . 33
S13 Comparison of the four subsampling methods for Iceland alignments . . . 34
S14 Comparison of the four subsampling methods for Taiwan alignments . . . 35
S15 Comparison of subsampling methods on SARS-CoV-2 introductions over time. . . . 36

List of Tables

S1 Sequencing protocols. 5
S2 Summary of alignments 7
S3 Epochs used in MTBD 8
S4 Parameters of sigmoid model for human movement 12
S5 Date boundaries of mobility reduction 12
S6 Summary of prior distributions 16
S7 Prior distributions for s 18
S8 Prior distributions for π_G 19
S9 Priors for m under SC 19
S10 Effective sample sizes under the MTBD model 21
S11 Effective sample sizes under the SC model 22
S12 Effective sample sizes under the DPG model 22
S13 Effective sample sizes under the DPG2 model 23
S14 Estimated number of imports and exports (“small-time”) 37
S15 Estimated number of imports and exports (“large-time”) 38
S16 Estimated number of imports and exports (“small-active”) 39
S17 Estimated number of imports and exports (“large-active”) 40

1 Viral genomic sequencing

RNA from 217 confirmed COVID-19 cases in New Zealand were obtained from diagnostic laboratories around the country. Viral RNA is reverse transcribed with SSIV with random hexamers, and then amplified using multiple overlapping PCR reactions spanning the viral genome, by employing Q5 HotStart High-Fidelity DNA Polymerase. Two primer schemes were used in this study: 1) ARTIC network protocol (V1 and V3) and 2) the New South Wales (NSW) primer set described in Eden et al. (2020) . Samples processed with the ARTIC protocol were sequenced on R9.4.1 MinION flow cells using the Oxford NanoPore ligation sequencing protocol (Loman et al., 2020). Sample processed with the NSW primer set were sequenced on Illumina NextSeq and MiSeq flowcells in paired-end 300 cycle format using the Nextera-XT library protocol. Table S1 describes the number of genomes processed using each protocol.

Table S1: Sequencing protocols used to sequence the 217 New Zealand SARS-CoV-2 genomes.

Protocol	Technology	Number of genomes
ARTIC V1	Oxford Nanopore	9
ARTIC V3	Oxford Nanopore	86
NSW	Illumina	122

For both methods, alignment to the reference genome MN908947.3 was followed by consensus calling for the major alleles on variation sites. Regions are masked with *N*'s in the final genome when amplicon failed to reach sufficient depth. Genomes with fewer than 3000 *N*'s in their consensus genome were used in the analysis presented here. The nanopore reads generated with Nanopore sequencing of ARTIC primer sets (V1 and V3) were mapped and assembled using the ARTIC bioinformatics pipeline (v1.1.0; Loman et al. 2020) with the "–medaka" flag enabled in the minion step. For the NSW primer set, raw reads were quality and adapter trimmed using `trimmomatic` (v0.36; Bolger et al. 2014) with the following settings: removing first 15 and last 20 bases of each read, minimum trailing quality of 30, 4nt-moving average quality of 25, and length >= 100bps.

Trimmed paired reads were mapped to reference using `bwa`. Primer sequences were masked using `iVar` (v1.2; Grubaugh et al. 2019). Duplicated reads were marked using `Picard` (v2.10.10; `http://broadinstitute.github.io/picard/`) and not used for SNP calling or depth calculation. Single nucleotide polymorphisms (SNPs) were called using `bcftools` mpileup (v1.9; `http://samtools.github.io/bcftools/`). SNPs within genomic regions amplified by each primer sets and primer regions were called separately. SNPs were quality trimmed using `vcffilter` (vcflib v1.0.0; Garrison 2014) requiring 20x depth and overall quality of 30. Positions that are less than 20x were masked as *N* in the final consensus genome. In addition, positions with an alternative allele frequency between 20 and 79% were also masked as *N*.

2 Sequence preprocessing

2.1 Filtering

Viral genomic sequences were filtered by observing the following constraints:

1. All viral sequences must have a human host;

2. All sequences must have date resolution down to the day of the month;

3. All sequences be more than 25 kb in length;

4. The proportion of ambiguous sites in a sequence must be less than 0.005 (appoximately 150 sites) (due to a shortage in sequences, samples from New Zealand and Taiwan are exempt from this constraint);

5. Sequences on the "NextStrain CoV exclude list" (Hadfield et al., 2018); URL: `https://github.com/nextstrain/ncov/blob/master/defaults/exclude.txt`) are excluded from the analysis. This list contains sequences which were identified as problematic either through manual inspection, or due to the sample falling outside 4 interquartile ranges from the mean root-to-tip distance (calculated using TreeTime; Sagulenko et al. 2018).

2.2 Subsampling

Because analysing all available genomic data using our Bayesian model is prohibitively slow, subsampling the full dataset is necessary. We assess four subsampling schemes producing one alignment each, differing with respect to (i) dataset size n (either "small" or "large"), and (ii) the method used to select sequences (either "time" or "active"). In (i), the rest-of-the-world deme ($\mathcal{RW}$) contributes 200 or 500 sequences to the "small" and "large" datasets, respectively. Out of these sequences, 40 (for "small") or 80 (for "large") always come from China for the phylogenetic signal they carry with respect to the root of the infection tree. The island deme ($\mathcal{IS}$) then contributes the remaining sequences to n, up to a maximum of 250. Specifically, Australia contributes 250 sequences, Iceland 250, New Zealand 217, and Taiwan 76 (see more details on our deme definitions below). In (ii), the method consists of either (a) randomly choosing one sequence from each of n dates sampled with replacement (i.e., picking uniformly through time; the "time" method), or (b) sampling (with replacement) n country-date pairs proportionally to the number of active cases reported in that country on that date (according to `https://www.worldometers.info/coronavirus/`), with one sequence chosen randomly from each pair (the "active" method).

2.3 Alignment

After filtering and subsampling sequences, alignments are generated using MAFFT under its default settings (Katoh et al., 2009), yielding a total of 16 alignments (four alignments per geographical model).

Table S2: Taxa count N, alignment length L, and A, C, G, T, and ambiguous (?) content are reported for each alignment used in this study.

Island	Alignment	N	L (kb)	A (%)	C (%)	G (%)	T (%)	? (%)
Australia	Small-time	450	30	29.7	18.3	19.5	32	0.1
Australia	Small-active	450	30	29.7	18.3	19.5	32	0.1
Australia	Large-time	750	30	29.7	18.3	19.5	31.9	0.1
Australia	Large-active	750	30	29.7	18.2	19.5	31.9	0.1
Iceland	Small-time	450	30	29.7	18.3	19.5	32	0.2
Iceland	Small-active	450	30	29.7	18.3	19.5	32	0.2
Iceland	Large-time	750	30	29.7	18.2	19.5	31.9	0.2
Iceland	Large-active	750	30	29.7	18.3	19.5	32	0.1
New Zealand	Small-time	417	30	29.5	18.1	19.4	31.7	0.9
New Zealand	Small-active	417	30	29.5	18.2	19.4	31.8	0.8
New Zealand	Large-time	717	30	29.5	18.1	19.4	31.7	0.5
New Zealand	Large-active	717	30	29.6	18.2	19.4	31.8	0.5
Taiwan	Small-time	310	30	29.5	18.1	19.4	31.7	0.1
Taiwan	Small-active	310	30	29.6	18.2	19.4	31.8	0.1
Taiwan	Large-time	610	30	29.5	18.1	19.4	31.7	0.1
Taiwan	Large-active	610	30	29.3	18	19.2	31.5	0.1

3 Demes and epochs

We consider four geographical models – each comprised of a target island deme $\mathcal{IS}$ (Australia, Iceland, New Zealand, or Taiwan), and a mutually exclusive "rest-of-the-world" deme $\mathcal{RW}$. The $\mathcal{RW}$ grouping of viral lineages is not biologically meaningful – it is a computational and modelling convenience – as its samples are further structured among themselves and not necessarily more similar to each other than to sequences from island demes. For this reason, the parameters associated with the $\mathcal{RW}$ deme are not interpreted and are treated as nuisance parameters.

Our MTBD analyses employ a general model implementation that allows for piecewise rate changes over the course of the infection (Kühnert et al., 2016), i.e., rates are held constant within time intervals, but are allowed to vary between them. An infection process is characterised by the time of the origin at t_0, and the end of its last (f-th) interval, t_f (which is also the last sampling time). Each of the f intervals is then defined by a boundary time $t_i \in t = (t_1, ..., t_{f-1})$, where $t_0 < t_1 < ... < t_{f-1} < t_f$. Given a parameter $p \in \{R_e, b, s, m\}$, p_i is the rate inside the i-th interval $[t_{i-1}, t_i)$. Time intervals t are both parameter-specific and deme-specific, and are treated as data (i.e., they are not sampled; Table S3).

Table S3: Time intervals (epochs) used by the MTBD model, specified as $[t_i,\ t_{i+1})$. t_0 and t_f correspond to the origin and the date the last sample was collected. All other times are deme-specific, and in the case of s, sample specific. The "First reported case" dates are in Table 1 of the main article. For simplicity, the "Border close" date is held at March 20 for all demes, despite a one day difference existing in practice. "Mobility decline" dates are shown in Table S5. The sampling proportion s is held constant at 0 from the start of the infection until the first tip within the deme (an alignment-specific date), and then estimated in the second epoch. As there is no migration signal before the first reported case within each island, the migration rate $m_{\mathcal{IS},\mathcal{RW}}$ is fixed to a negligible quantity (10^{-6}) due to its non-identifiability. Nuisance parameters ($\mathcal{RW}$ deme) are modelled with a single time interval over which they are sampled.

Parameter(s)	Deme	i	Time interval<br>Start (t_i)	 End (t_{i+1})
R_e and b	$\mathcal{IS}$	0	t_0	First reported case
R_e and b	$\mathcal{IS}$	1	First reported case	Mobility decline
R_e and b	$\mathcal{IS}$	2	Mobility decline	t_f
R_e and b	$\mathcal{RW}$	0	t_0	t_f
$s = 0$	$\mathcal{IS}$	0	t_0	First sample
s	$\mathcal{IS}$	1	First sample	t_f
$s = 0$	$\mathcal{RW}$	0	t_0	First sample
s	$\mathcal{RW}$	1	First sample	t_f
$m_{\mathcal{IS},\mathcal{RW}} = 10^{-6}$	–	0	t_0	First reported case
$m_{\mathcal{RW},\mathcal{IS}}$	–	0	t_0	First reported case
$m_{\mathcal{IS},\mathcal{RW}}$	–	1	First reported case	Border close
$m_{\mathcal{RW},\mathcal{IS}}$	–	1	First reported case	Border close
$m_{\mathcal{IS},\mathcal{RW}}$	–	2	Border close	t_f
$m_{\mathcal{RW},\mathcal{IS}}$	–	2	Border close	t_f

3.1 A model of human movement decrease for mobile phone data

Defining time intervals characterised by different levels of human movement is challenging, as the four "island" considered here differ markedly in their response to COVID-19. No single date (such as the day of lockdown, for example) could be used across countries: while the governments of New Zealand and Australia locked human movement down to different extents for several weeks, those of Iceland and Taiwan, on the other hand, found it sufficient to restrict the size of gatherings and encourage social distancing (Hale et al., 2020).

In order to define country-specific time intervals in a statistically principled way, we first parameterise human movement decrease with a sigmoid model describing how mobility (Apple, 2020; Fig. S1) changes as a function of time t:

$$s(t|t_0, d, a, r) = 1.0 - a\big(S(mt + c) - S(m_r t + c_r)\big), \tag{1}$$

the parameters of which are described in table S4. Here, S corresponds to the sigmoid (logistic) function $S(x) = \frac{1}{1+e^{-x}}$, and m, c, m_r and c_r are deterministically defined in terms of other parameters such that:

1. $1 - S\big(m(t_0 + d) + c\big) = S\big(mt_0 + c\big) = \frac{1-\alpha}{2}$, i.e., α of the decrease occurs between t_0 and $t_0 + d$,
2. $S\big(m_r(t_0 + d) + c_r\big) = \frac{1-\alpha}{2}$, i.e., only $\frac{1-\alpha}{2}$ of the recovery has occurred at the end of the decrease, and
3. $S\big(m_r(t_0 + d + d_r) + c_r\big) = r$, i.e., r of the recovery has occurred d_r after the end of the decrease.

We apply Equation 1 to our set of four demes $\mathcal{I} = \{1, 2, 3, 4\}$ by allowing each country $i \in \mathcal{I}$ to have its own parameters controlling the timing of the decrease, t_{0i} and d_i. Additionally, we can consider the three modes of transportation j individually (i.e., $j \in \mathcal{J} = \{1, 2, 3\}$), and allow each (country i–mode of transportation j) pair to be characterised by its own decrease amplitude, a_{ij}, recovery amplitude r_{ij}, and mobility standard deviation parameter ν_{ij} (Table S4). Finally, we let each combination of country, mode of transportion, and weekday k have its own baseline mobility mean of b_{ijk}. Note that these considerations make all parameters in equation 1 multidimensional (Table S4).

We model the human movement underlying mobility data $\mathbf{M} = (m_{ijk})$ (i-th country, j-th mode of transportation, k-th weekday) by assuming M_{ijk} is sampled from a Normal distribution, letting:

$$f(M_{ijk}|b_{ijk}, \nu_{ij}, t_{0i}, d_i, a_{ij}, r_{ij}) = \mathcal{N}(M_{ijk}|b_{ijk} \times s(t; t_{0i}, d_i, a_{ij}, r_{ij}), \nu_{ij}), \tag{2}$$

where $\mathcal{N}(\cdot|\mu, \sigma)$ is the probability density function of a normal distribution with mean μ and standard

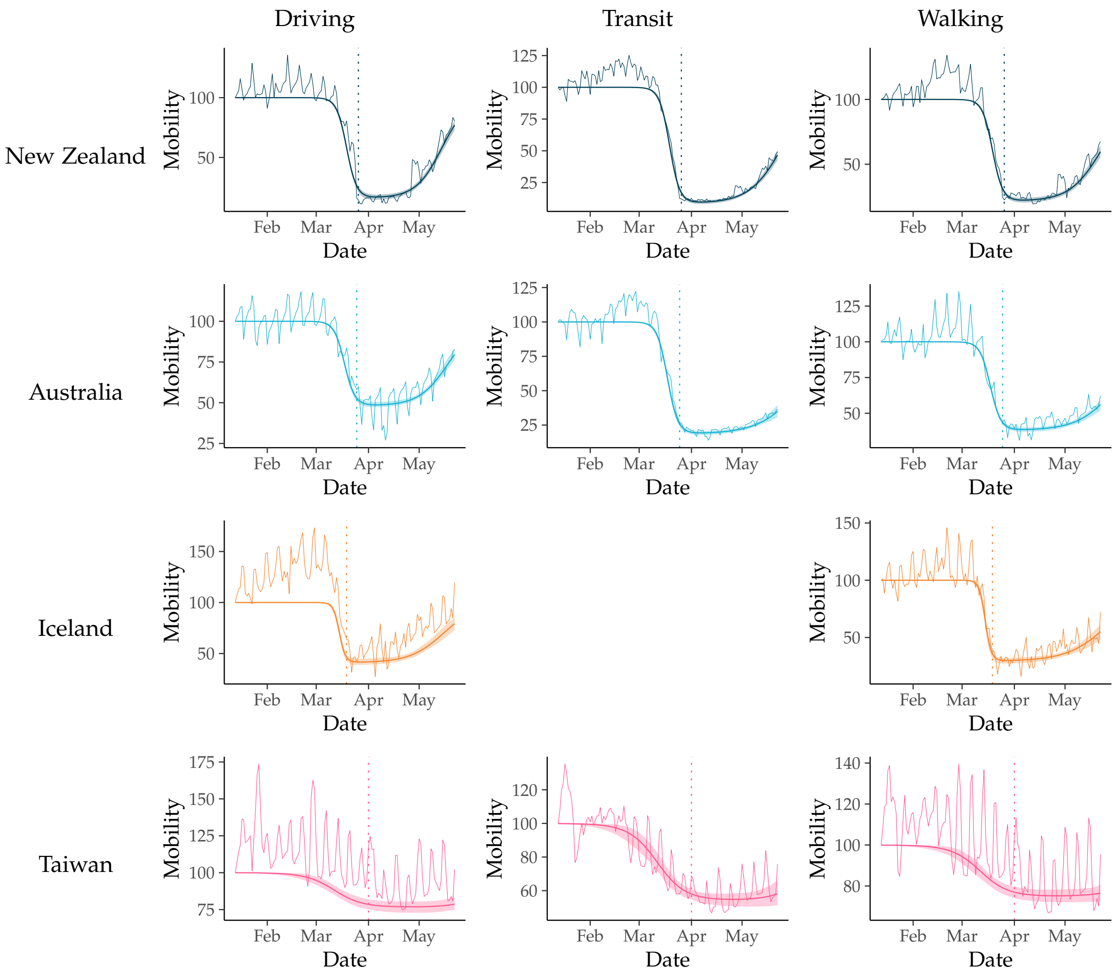

Fig. S1: Cell phone mobility data from New Zealand, Australia, Iceland and Taiwan, for three kinds of transportation: driving, transit and walking. The "wiggly" lines correspond to the raw data. Fit sigmoid models are shown in all graphs as a smooth shaded curve (the 2.5–97.5% quantile interval) and a solid line inside of it (the sigmoid function).

deviation σ. Note that the baseline mean of this distribution, b_{ijk}, is scaled by the sigmoid function $s(\cdot)$ describing the mobility decrease effect in equation 1.

Our full model is hierarchical and allows the mobility signal of the different nations to jointly and mutually inform all model parameters. This is done by using hierarchical priors, and estimating shared hyperparameters $\theta = \{\mu_{t0}, \sigma_{t0}, \mu_d, \sigma_d, \alpha_{aj}, \beta_{aj}, \alpha_{rj}, \beta_{rj}, b_{ijk}, \nu_{ij}\}$. The unnormalised posterior density of our full model is given by:

$$f(\boldsymbol{b}, \boldsymbol{\nu}, \boldsymbol{t_0}, \boldsymbol{d}, \boldsymbol{a}, \boldsymbol{r}, \theta | M) \propto f(\theta) \prod_{i \in \mathcal{I}} f(t_{0i}, d_i | \theta) \prod_{j \in \mathcal{J}_i} f(a_{ij}, r_{ij}, \nu_{ij} | \theta) \tag{3}$$

$$\prod_{k=1}^{7} \Big(f(b_{ijk} | \theta) \Big) \prod_{t \in M_{ij}} f(M_{ij\omega(t)} | b_{ij\omega(t)}, \nu_{ij}, t_{0i}, d_i, a_{ij}, r_{ij}), \tag{4}$$

where $\omega(t)$ is a deterministic function that returns the weekday of a time point t, $f(\theta)$ is the hyperprior density on hyperparameters θ, and $f(t_{0i}, d_i|\theta)$, $f(a_{ij}, r_{ij}, \nu_{ij}|\theta)$ and $f(b_{ijk}|\theta)$ are the prior densities on country-, transportation mode-, and weekday-level parameters, respectively (Table S4). We chose our priors following recommendation in Gelman et al. (2006). Priors are listed table S4.

We determined the boundary between time intervals characterised by "high" and "low" human movement using New Zealand as a reference. As a post processing step after MCMC, we computed and logged the proportion of mobility that had gone down in New Zealand by 26 March 2020 (according to the fitted sigmoid model), and then deterministically recorded the dates by which the other islands had undergone the same proportional reduction. Let $\mathcal{S}(t|t_0, d) = S(mt + c)$, the sigmoid function describing the decrease in mobility, where m and c are defined as in Equation 1. Let $\mathcal{S}^{-1}(p|t_0, d)$ be the inverse of this function. Then, for a given set of parameter values, the boundary date for country i is:

$$\mathcal{S}^{-1}(\mathcal{S}(26\ \text{March}|t_{0\text{z}}, d_\text{z})|t_{0i}, d_i),$$

where the subscript z refers to the index of New Zealand in the set $\mathcal{I}$. We used the posterior mean of this date as the boundary between time intervals.

Our model is implemented in PyMC3 (Salvatier et al., 2016), and we employed the No-U-Turn Sampler (Hoffman and Gelman, 2014) to sample the posterior distribution. We ran three independent chains and sampled each 10000 times (after 1000 tuning samples). The three chains were visually checked for convergence and combined, yielding effective samples sizes larger than 200.

Table S4: Parameters of sigmoid model for human movement. These parameters share names with random variables from other models, but represent distinct quantities only used in the sigmoid model. N_o indicates a bounded Normal distribution conditioned on non-negativity, and HC indicates a Half-Cauchy distribution with location 0 and conditioned on non-negativity.

Parameter	Description	Prior
	**Mobility model parameters**	
$\mathbf{t_0}$	$\mathbf{t_0} = (t_{0i})$, $i \in \mathcal{I}$, the start of the mobility decrease effect in country i	$N(\mu_{t_0}, \sigma_{t_0})$
$\mathbf{d}$	$\mathbf{d} = (d_i)$, $i \in \mathcal{I}$, the duration of the mobility decrease effect in country i	
$\mathbf{a}$	$\mathbf{a} = (a_{ij})$, $i \in \mathcal{I}$, $j \in \mathcal{J}$, the proportional amplitude of the mobility decrease for country i and transportation mode j	$\text{Beta}(\alpha_{aj}, \beta_{aj})$
$\mathbf{r}$	$\mathbf{r} = (r_{ij})$, $i \in \mathcal{I}$, $j \in \mathcal{J}$, the proportion of mobility decrease that recovers by $(t_0 + d + d_r)$ in country i, for transporation mode j	$\text{Beta}(\alpha_{rj}, \beta_{rj})$
$\boldsymbol{b}$	$\boldsymbol{b} = (b_{ijk})$, $i \in \mathcal{I}$, $j \in \mathcal{J}$, $k \in \mathcal{K}$, the baseline mobility for country i, transportation mode j and weekday k	$N_o(\mu_{bjk}, \sigma_{bjk})$
$\boldsymbol{\nu}$	$\boldsymbol{\nu} = (\nu_{ij})$, $i \in \mathcal{I}$, $j \in \mathcal{J}$, the mobility standard deviation for country i and transportation mode j	HC(0.1)
α	Proportion of decrease effect which occurs between t_0 and $t_0 + d$	Fixed to 0.99
d_r	Duration over which recovery is measured	Fixed to 60
	**Hyperparameters**	
μ_{t_0}	$\mathbf{t_0}$ prior mean	N(Mar 25, 14 days)
σ_{t_0}	$\mathbf{t_0}$ prior standard deviation	HC(2.0)
μ_d	$\mathbf{d}$ prior mean	N_o(14 days, 7 days)
σ_d	$\mathbf{d}$ prior standard deviation	HC(2.0)
$\boldsymbol{\alpha_a}$	$\boldsymbol{\alpha_a} = (\alpha_{aj})$, $j \in \mathcal{J}$, $\mathbf{a}$ prior shape	N_o(2.0, 2.0)
$\boldsymbol{\beta_a}$	$\boldsymbol{\beta_a} = (\beta_{aj})$, $j \in \mathcal{J}$, $\mathbf{a}$ prior rate	N_o(2.0, 2.0)
$\boldsymbol{\alpha_r}$	$\boldsymbol{\alpha_r} = (\alpha_{rj})$, $j \in \mathcal{J}$, $\mathbf{r}$ prior shape	N_o(1.5, 2.0)
$\boldsymbol{\beta_r}$	$\boldsymbol{\beta_r} = (\beta_{rj})$, $j \in \mathcal{J}$, $\mathbf{r}$ prior rate	N_o(2.0, 2.0)
$\boldsymbol{\mu_b}$	$\boldsymbol{\mu_b} = (\mu_{bjk})$, $j \in \mathcal{J}$, $k \in \mathcal{K}$, $\boldsymbol{b}$ prior mean	N_o(1.0, 0.2)
$\boldsymbol{\sigma_b}$	$\boldsymbol{\sigma_b} = (\sigma_{bjk})$, $j \in \mathcal{J}$, $k \in \mathcal{K}$, $\boldsymbol{b}$ prior standard deviation	HC(1.0)

Table S5: Date boundaries of mobility reduction, according to the model described in Section 3.1 which was fit to mobile phone data (Apple, 2020). All dates are within the year of 2020. The start dates were used as epoch boundaries for R_e and b.

$\mathcal{IS}$	Start	Mean	End
New Zealand	Mar 26	Mar 26	Mar 26
Australia	Mar 24	Mar 25	Mar 26
Iceland	Mar 18	Mar 19	Mar 20
Taiwan	Mar 27	Mar 31	Apr 05

4 Model definition

4.1 Substitution and clock models

In all analyses, the phylogenetic likelihood was evaluated under the HKY substitution model with estimated nucleotide equilibrium frequencies π_D. Sites were partitioned into non-coding sites and the three codon positions, with each partition having its own substitution model parameters. The end regions were masked because they are suspected to harbour many sequencing errors (as described by `http://virological.org/t/issues-with-sars-cov-2-sequencing-data/473`). Because SARS-CoV-2 genomes have undergone a small number of mutations (Lai et al., 2020; Li et al., 2020b; Rambaut, 2020), we assumed a strict molecular clock in all analyses.

4.1.1 Partition schemes and substitution model selection

We compared substitution models and partition schemes based on their posterior distributions over $\mathcal{T}$, and picked the simplest model generating the least different posterior distribution. For example, substitution models more complex than HKY did not affect the posterior distribution of $\mathcal{T}$ relative to HKY, while simpler models such as JC69 (Jukes Cantor 1969) yielded a posterior distribution that was substantially different. Therefore, we selected HKY with estimated frequencies. Model comparison was done with BModelTest (Bouckaert and Drummond, 2017). By the same token, we did not opt for single partition analyses – which led to very different tree posteriors compared to the chosen partition scheme (see main text) – and rejected adding further partitions (e.g., on gene boundaries) because they did not alter the tree posterior significantly.

4.2 Phylodynamic models

4.2.1 Discrete phylogeography (DPG)

The DPG model (Lemey et al., 2009) employs a continuous-time Markov chain in similar fashion to substitution models used in molecular evolution studies, but one in which a single "character" is considered: the discrete location (the deme) a lineage occupies in space. Lineages are allowed to change demes over time, with demes being inherited by children lineages according to a phylogenetic tree. As opposed to nucleotide substitution models that emit four discrete states, however, the DPG model can emit d states, where d is the number of demes represented in the data.

Under the DPG model, state transitions correspond to migrations events (i.e., a lineage moves from one

deme to another) that happen as described by a 2×2 symmetric, infinitesimal rate matrix:

$$\mathbf{m} = \mu_m \mathbf{S\Pi} = \mu_m \begin{bmatrix} -\pi_{\mathcal{IS}} & \pi_{\mathcal{IS}} \\ \pi_{\mathcal{RW}} & -\pi_{\mathcal{RW}} \end{bmatrix}, \tag{5}$$

where μ_m is an overall rate scaler, $\mathbf{S}$ is a matrix of relative rates of changing demes, and $\mathbf{\Pi} = \text{diag}(\pi)$ (with π being the equilibrium deme frequencies; Table S6). Note that (i) $\mathbf{\Pi}$ is estimated as opposed to $\mathbf{S}$, because the two are non-identifiable (i.e., a symmetric model), (ii) $\mathbf{\Pi}$ is normalised such that μ_m reflects the number of migration events per unit time (Lemey et al., 2009), and (iii) the frequencies $\mathbf{\Pi}$ at the root are fixed at $\mathbf{\Pi} = (\pi_{\mathcal{IS}}, \pi_{\mathcal{RW}}) = (0, 1)$ to incorporate knowledge of the infection originating outside of the four island demes of interest.

Finally, we couple our DPG model implementation to the Bayesian skyline model (Drummond et al., 2005) to allow for effective population size (N_e) changes over ten time intervals. Following the notation from the main manuscript, under the DPG we have:

$$f(\mathcal{T}|\theta_\tau) = f(\mathcal{T}|\mu_m, \pi, N_e). \tag{6}$$

4.2.2 Two epoch discrete phylogeography (DPG2)

The two epoch discrete phylogeography model (DPG2) generalises the DPG model by distinguishing two time intervals: one before and one after t =19 March 2020, the date in which both New Zealand and Taiwan closed its borders (Australia and Iceland closed theirs in the following day). These two time intervals are characterised by their own $\mathbf{m}$ rate matrix. Following the notation from the main manuscript, under the DPG2 model we have:

$$f(\mathcal{T}|\theta_\tau) = f(\mathcal{T}|\mu_m, \pi, N_e, t). \tag{7}$$

Note that t here is fixed and treated as data. In computing this density, only one $\mathbf{m}$ matrix is used for branches contained in their entirety within a time interval; branches that are intersected by t, on the other hand, have transition probabilities computed by combining two different $\mathbf{m}$ matrices (Bielejec et al., 2014).

4.2.3 Structured coalescent (SC)

The fourth phylodynamic model we used was the structured coalescent model implemented in MASCOT (**M**arginal **A**pproximation of the **S**tructured **Co**alescen**T**; Müller et al. (2018)). Structured coalescent models allow one to estimate demographic parameters and genealogical relationships among sub-populations (demes), but estimation under exact implementations is costly because lineage ancestral states must be sampled with MCMC. The marginal approximation of the structured coalescent (MASCOT) model, on the other hand, circumvents this issue by integrating over all possible migration histories (Müller et al., 2018). Under this model, we estimate population sizes for each deme i, $\mathbf{N} = (N_e^i)$, and an among-deme migration rate matrix $\mathbf{m}$. Following the notation from the main text:

$$f(\mathcal{T}|\theta_\tau) = f(\mathcal{T}|S, m, \mathbf{N}), \tag{8}$$

where $\mathcal{S}$ is treated as data containing the deme information for each sample.

4.2.4 Multi-type birth-death (MTBD)

The full probabilistic graphical model given the MTBD tree prior used in this study can be seen in Fig. S2.

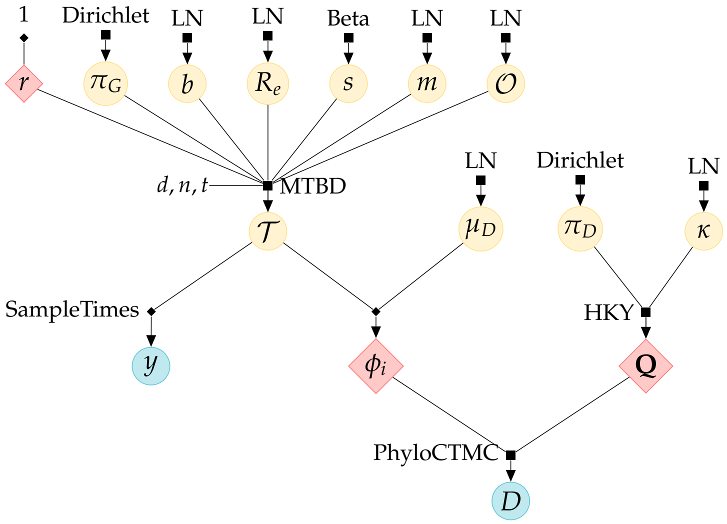

Fig. S2: Full MTBD probabilistic graphical model used in our study. Yellow and blue circles correspond to parameters and observed data, respectively. Red diamonds are deterministic functions and their outcomes. Filled squares represent sampling distributions (e.g., "MTBD", multitype birth-death; "HKY", Hasegawa-Kishino-Yano substitution model).

4.3 Prior distributions

The prior distributions used in this article are summarised in Table S6. Priors for $\mathcal{O}$, s, m, and π_G were programatically generated for each alignment and are detailed in the following subsections.

Table S6: Summary of prior distributions. $\Gamma(\alpha, \beta)$ denotes a gamma distribution with shape parameter α and inverse scale parameter β; "LN(μ, σ)" denotes a log-normal distribution with log-space mean μ and log-space standard deviation σ; "Exp(μ)" denotes an exponential distribution with a mean of μ. "Dir($\alpha_1, \ldots, \alpha_m$)" is a Dirichlet distribution with shapes $(\alpha_1, \ldots, \alpha_m)$. *Origin time prior distribution has an offset (see Section 4.3.1). **s is parameterised as s' (see Section 4.3.2).

Parameter	Description	Prior distribution
	**DPG/DPG2 parameters**	
μ_m	Migration rate scaler	$\Gamma(0.001, 1000)$
π	Equilibrium deme frequencies	$\mathrm{Dir}(1, 1)$
$N_e(0)$	Effective population size baseline	$\mathrm{LN}(0, 2)$
$N_e(i), i > 0$	Effective population size change	$\mathrm{Exp}(N(i-1))$
	**SC parameters**	
N_e	Effective population size	$\mathrm{LN}(0.007, 1)$
m	Migration rates	
	**MTBD parameters**	
R_e	Basic reproduction number	$\mathrm{LN}(1.0, 0.7)$
b	Rate of becoming non-infectious	$\mathrm{LN}(4.09, 0.2)$
$\mathcal{O}$	Origin time	LN(-1.98, 0.4)*
r	Removal probability	1
s	Sampling proportion	$\mathrm{Beta}(1.1, 8.0)$**
m	Migration rates	$\mathrm{LN}(0, 1)$
π_G	Equilibrium deme frequencies (see Section 4.3.3)	
	**Substitution model parameters (shared)**	
κ	HKY transition-transversion ratio	$\mathrm{LN}(1, 1.25)$
π_D	HKY nucleotide frequencies	$\mathrm{Dir}(1, 1, 1, 1)$
μ_D^1	Molecular clock rate (for DPG and DPG2)	$\mathrm{LN}(-7, 1.25)$
μ_D^2	Molecular clock rate (for MTBD and SC)	$\mathrm{LN}(-7, 0.25)$

4.3.1 Prior for origin time

As defined in the main text, origin time $\mathcal{O}$ represents the height of the sampled infection tree ($\mathcal{T}$) in years, which goes from the most recent sample to patient zero, the first case of the disease. The time of the most recent sample can vary depending on the sampling scheme (Section 2), but with the exception of one alignment, all alignments had their most recent sample taken on 29 April 2020. Patient zero has been only tentatively placed on 17 November 2020 in Hubei, China, but at least 60 cases had been confirmed with certainty by 20 December in that country (see `https://www.scmp.com/news/china/society/article/3074991/coronavirus-chinas-first-confirmed-covid-19-case-traced-back`).

We assumed an offset log-normal prior distribution for $\mathcal{O}$; specifically, $(\mathcal{O} - \delta) \sim \text{LN}(\mu = -1.98, \sigma = 0.4)$, where δ is an offset defined by the time interval between the alignment-dependent first sample and 20 December 2020, a day we judged to provide positive evidence COVID-19 was spreading in China. By applying variable offsets, one can use the same log-normal parameterisation (i.e., same mean and standard deviation) for all alignments. Therefore, irrespective of the subsampling scheme, this prior translates into the first case having its mean and modal occurrence dates on 26 October and 7 November 2020, respectively. This prior also implies that the first case of COVID-19 has (1) zero probability of having happened after 20 December 2019, and (2) asymptotically zero probability of happening before mid 2019 (Fig. S3).

4.3.2 Priors for sampling proportion s

Under the MTBD model, $s = \frac{\psi}{\psi+\mu}$ describes the proportion of sampled individuals out of all sampled and removed individuals. In the time interval prior to the first sample no sampling has happened, and hence $s = 0$. After the first sample, s has an upper-limit u such that $s \leq u$:

$$u = \frac{\text{Number of samples}}{\text{Number of confirmed cases as of date } t_f}. \tag{9}$$

The denominator on the right-hand side comes from the assumption that once an individual has become a confirmed case, they are removed from the infectious pool (i.e., we assume $r = 1$). If a large proportion of infections are asymptomatic or mildly symptomatic, as is the case of COVID-19 (Day, 2020b,a; Li et al., 2020a; Lu et al., 2020), s can be an order of magnitude smaller than u.

In the equation above, note that u can be specific to a deme d, in which case we only count individuals from deme d when calculating u_d. u_d is also alignment specific; for example, in the "small-active" alignment (see Section 2) where New Zealand is the target island, $u_{\mathcal{IS}} = 0.147$ and $u_{\mathcal{RW}} = 8.22 \times 10^{-5}$ (3 sf). The full set of values are available in the GitHub repository accompanying this article. The number of confirmed COVID-19 cases was compiled by `https://www.worldometers.info/coronavirus/`, and we programmatically accessed these figures from the Worldometers Daily Data GitHub repository by David Bumbeishvili (`https://github.com/bumbeishvili/covid19-daily-data`).

We reparameterised s as $s' = \frac{s}{u_d} \in [0, 1]$ so that a natural prior choice for s' would be a Beta distribution. More specifically, we assumed $s' \sim \text{Beta}(\alpha = 1.1, \beta = 8.0)$, which sets the mean of s at $0.12 u_d$ and bounds it at 0.0 and u_d.

Table S7: Alignment-specific upper limits for s in the MTBD model. Rounded to 3 sf.

Alignment	Island $\mathcal{IS}$	$u_{\mathcal{IS}}$	$u_{\mathcal{RW}}$
Small-active	Australia	0.0373	8.62×10^{-5}
	Iceland	0.139	8.98×10^{-5}
	New Zealand	0.147	8.22×10^{-5}
	Taiwan	0.255	8.52×10^{-5}
Small-time	Australia	0.0369	7.61×10^{-5}
	Iceland	0.138	8.83×10^{-5}
	New Zealand	0.147	8.32×10^{-5}
	Taiwan	0.255	7.44×10^{-5}
Large-active	Australia	0.0372	1.98×10^{-4}
	Iceland	0.14	1.96×10^{-4}
	New Zealand	0.147	2.02×10^{-4}
	Taiwan	0.255	1.91×10^{-4}
Large-time	Australia	0.0369	1.89×10^{-4}
	Iceland	0.138	1.93×10^{-4}
	New Zealand	0.147	1.8×10^{-4}
	Taiwan	0.255	1.92×10^{-4}

4.3.3 Priors for MTBD geographical frequencies π_G

Under our MTBD model, the equilibrium frequency of each deme is assumed to be proportional to that deme's human population H_d. In the case of $\mathcal{RW}$, H_d is the sum of the population of countries represented in an alignment, so this quantity is thus dependent on the subsampling scheme. π_G is given by:

$$\pi_G = (\pi_G(\mathcal{IS}),\ \pi_G(\mathcal{RW})) \sim \text{Dirichlet}\Big(\frac{H_{\mathcal{IS}}}{H_{\mathcal{IS}} + H_{\mathcal{RW}}} \times k_{\pi_G},\ \frac{H_{\mathcal{RW}}}{H_{\mathcal{IS}} + H_{\mathcal{RW}}} \times k_{\pi_G}\Big). \tag{10}$$

Scalar k_{π_G} controls the variance and was set to $k_{\pi_G} = 10^4$. The full set of priors for π^G is presented in Table S8.

Table S8: Alignment-specific priors for π_G in the MTBD model. "Dir" stands for Dirichlet.

Alignment	Island $\mathcal{IS}$	Prior of π_G
Small-active	Australia	Dir(68.2, 9938.1)
	Iceland	Dir(1, 9999)
	New Zealand	Dir(20.6, 9979.4)
	Taiwan	Dir(66.8, 9933.2)
Small-time	Australia	Dir(58.8, 9941.2)
	Iceland	Dir(0.9, 9999.1)
	New Zealand	Dir(11.9, 9988.1)
	Taiwan	Dir(53.3, 9946.7)
Large-active	Australia	Dir(63.6, 9936.4)
	Iceland	Dir(0.9, 9999.1)
	New Zealand	Dir(13, 9987)
	Taiwan	Dir(60.1, 9939.9)
Large-time	Australia	Dir(55, 9945)
	Iceland	Dir(0.8, 9999.2)
	New Zealand	Dir(10.5, 9989.5)
	Taiwan	Dir(54, 9946)

Table S9: Alignment-specific priors for m in the SC model for the "small-active" method. "LN" is a LogNormal distribution.

Island $\mathcal{IS}$	$m_{\mathcal{RW},\mathcal{IS}}$	$m_{\mathcal{IS},\mathcal{RW}}$
Australia	LN(−15.1, 1)	LN(−4.7, 1)
Iceland	LN(−19.6 1)	LN(−0.8, 1)
New Zealand	LN(−16.8, 1)	LN(−3.4, 1)
Taiwan	LN(−14.9, 1)	LN(−4.9, 1)

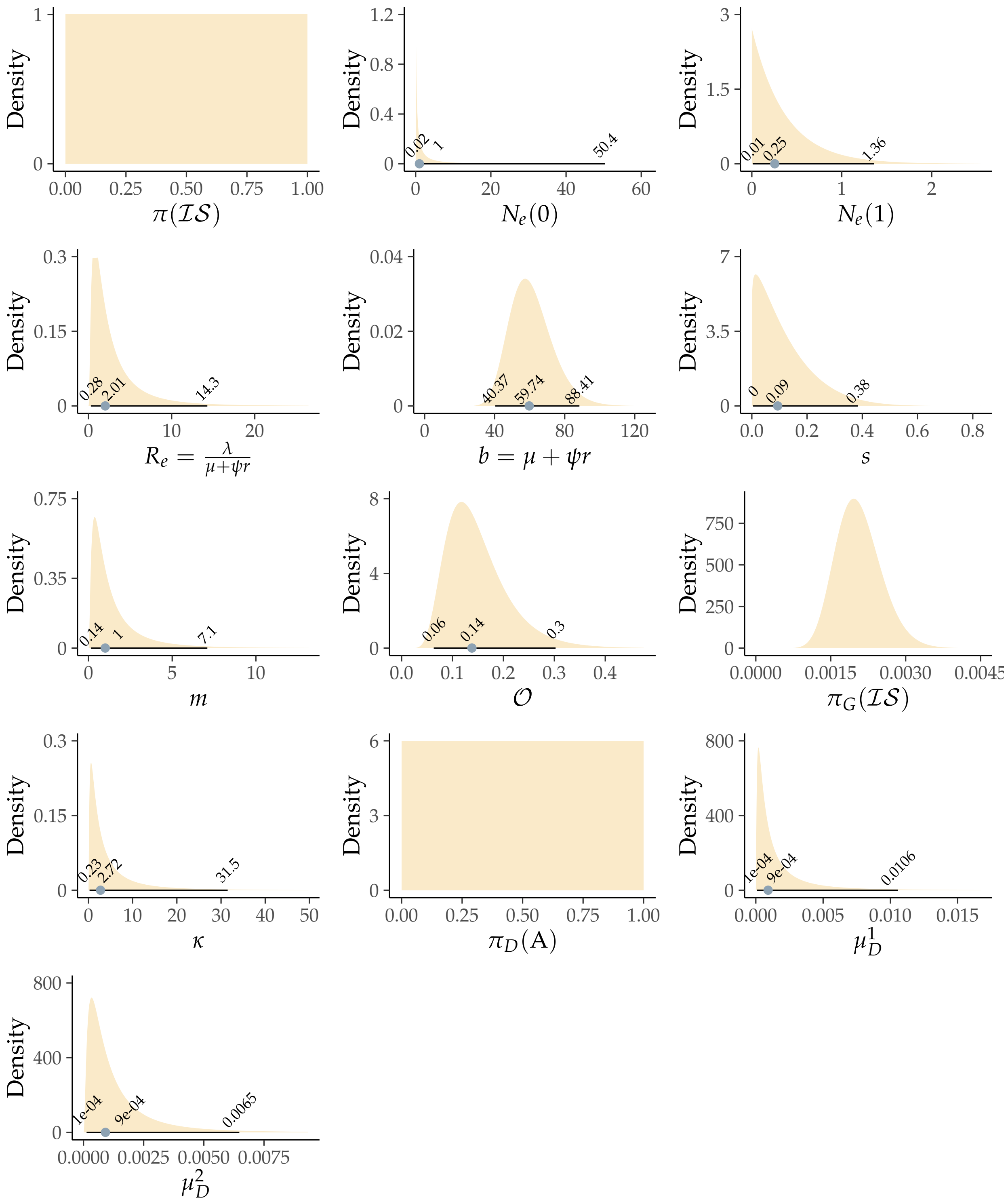

Fig. S3: Prior probability distributions used in our models for phylodynamic analyses. Parameters appear in the same order as in Table S6. Black horizontal lines indicate the exact intervals between 2.5% and 97.5% quantiles for the different priors; the blue dot indicates the 50% quantile (Dirichlet priors are multivariate distributions with non-unique quantiles, so those are not shown for these priors). The prior for $N_e(1)$ assumes $N_e(1) \sim \text{Exp}(\text{E}[N_e(0)])$. Only one dimension of each Dirichlet priors is shown. These dimensions are: $\pi(\mathcal{IS}) = 1 - \pi(\mathcal{RW})$ for MTBD geographical frequencies π; $\pi_G(\mathcal{IS}) = 1 - \pi_G(\mathcal{RW})$ for DPG geographical frequencies π_G; and $\pi_D(\text{A}) = 1 - \pi_D(\text{C}) - \pi_D(\text{G}) - \pi_D(\text{T})$ for nucleotide frequencies π_D.

5 Model implementation and parameter inference

All models used in this study are implemented in BEAST 2.6 (Bouckaert et al., 2019). Parameter inference is carried out using the Metropolis-Hastings algorithm, which generates a Markov chain that explores the posterior distribution by Monte Carlo simulation (MCMC). We thus employed MCMC to sample $(\mathcal{T}, \mu_c, \theta_\tau, \theta_s) \sim f(\mathcal{T}, \mu_c, \theta_\tau, \theta_s | D, y)$. We used a combination of MCMC and coupled MCMC (MC3; Müller and Bouckaert (2019)), as determined by examining either method's performance on a chain-by-chain basis.

Chain convergence was evaluated by observing a minimum ESS of 200 for the posterior, likelihood, and prior densities, as well as for all reported parameters (Tables S10, S11, S12, S13), with phylogenetic tree convergence inferred from a high correlation between posterior clade probabilities from independent chains (Fig. S4). Due to computational limitations, the "large" alignments did not fully converge for the MTBD or SC methods, and neither did the "small-active" method for MTBD. The results presented in the main text are not derived from the aforementioned chains. This highlights the appeal in the DPG model.

Table S10: Effective sample sizes (ESS) under the MTBD model. ESSes are in bold if they are problematic (i.e., less than 100). For vector parameters (such as κ, R_e, and b), the minimum ESS is reported.

Alignment	Island	Chain length (10^6)	posterior	likelihood	prior	μ	Height	κ	R_e	b	m	s	π_G
Large-active	New Zealand	93	158	320	170	187	355	2911	**19**	**24**	**19**	1135	4661
Large-active	Australia	110	**31**	214	**34**	**37**	129	3212	**18**	**49**	**10**	1175	4942
Large-active	Iceland	234	104	104	105	162	1909	3271	742	294	546	1734	11703
Large-active	Taiwan	162	277	411	238	345	360	4984	2075	2179	2309	2188	8091
Large-time	New Zealand	91	100	101	117	145	404	3101	**23**	**11**	471	971	3956
Large-time	Australia	122	**65**	211	**79**	195	1365	3823	224	**42**	130	1830	5910
Large-time	Iceland	218	**41**	110	**44**	**74**	116	6948	**5**	**5**	**5**	620	10901
Large-time	Taiwan	137	285	452	279	435	427	4779	1250	**30**	1597	3181	6322
Small-active	New Zealand	158	**70**	1497	**68**	**70**	**98**	2599	1208	488	125	940	7496
Small-active	Australia	122	**42**	123	**43**	**39**	**59**	3036	227	243	262	333	5600
Small-active	Iceland	374	173	1716	162	179	452	7905	1558	1083	434	9399	18679
Small-active	Taiwan	268	376	3165	355	320	444	9041	147	1034	143	8007	13204
Small-time	New Zealand	174	291	1930	396	414	976	5593	812	1787	840	6360	8243
Small-time	Australia	214	413	2683	397	573	1656	8106	2820	1049	1496	6411	10693
Small-time	Iceland	409	325	1030	295	404	1291	12747	265	684	159	14746	20189
Small-time	Taiwan	280	587	2128	533	730	2224	10535	1637	628	1957	7266	14023

Table S11: Effective sample sizes under the SC model. See Table S10 caption for notation.

Alignment	Island	Chain length (10^6)	posterior	likelihood	prior	μ	Height	κ	N_e	m
Large-active	New Zealand	269	121	**26**	211	310	181	5289	269	1703
Large-active	Australia	204	343	**91**	294	403	118	15768	379	1364
Large-active	Iceland	240	321	**31**	**72**	171	292	17602	106	496
Large-active	Taiwan	278	570	163	422	420	157	22350	536	1897
Large-time	New Zealand	269	351	**43**	290	266	381	21108	240	546
Large-time	Australia	317	668	340	407	806	299	26585	787	1782
Large-time	Iceland	253	441	195	374	675	305	20296	553	1936
Large-time	Taiwan	279	649	122	437	574	342	18439	426	1332
Small-active	New Zealand	270	331	**66**	1055	2036	804	16208	1612	3029
Small-active	Australia	240	833	334	691	1054	445	16094	1040	2771
Small-active	Iceland	240	1143	1106	1006	1953	560	12543	1650	2298
Small-active	Taiwan	270	1240	175	378	556	720	20527	557	4134
Small-time	New Zealand	270	770	669	526	217	668	11967	203	634
Small-time	Australia	240	768	418	408	986	334	16274	818	2333
Small-time	Iceland	240	820	158	249	604	1092	14314	344	1236
Small-time	Taiwan	270	1432	1735	835	1136	1687	15362	1111	2972

Table S12: Effective sample sizes under the DPG model. See Table S10 caption for notation.

Alignment	Island	Chain length (10^6)	posterior	likelihood	prior	μ	Height	κ	N_e	m	π
Large-active	New Zealand	577	125	173	134	125	306	2523	**75**	986	443
Large-active	Australia	1168	318	487	249	224	4269	5307	**80**	2274	853
Large-active	Iceland	967	1091	**47**	380	538	2759	4359	**10**	1238	1094
Large-active	Taiwan	1383	394	1270	311	229	611	4933	**86**	367	346
Large-time	New Zealand	545	259	101	277	607	2762	2469	**66**	146	144
Large-time	Australia	1070	875	848	668	1132	1921	5183	158	4860	3606
Large-time	Iceland	781	843	930	491	911	4226	4058	**89**	1448	1106
Large-time	Taiwan	1329	463	790	365	370	1658	6213	179	3963	2837
Small-active	New Zealand	2388	4405	5705	3986	5443	9610	7447	2672	9809	7970
Small-active	Australia	2307	3999	4945	3647	2826	8699	6068	1872	8931	7074
Small-active	Iceland	2085	923	1550	721	1815	5760	8442	825	6881	5364
Small-active	Taiwan	2700	4425	10601	3814	2723	2995	11254	3275	825	768
Small-time	New Zealand	2039	3740	5947	3605	4391	8431	6182	1409	7195	5130
Small-time	Australia	2275	3482	2367	2331	2197	23924	7472	2720	12194	10330
Small-time	Iceland	1353	1816	513	481	597	17151	6428	1860	4062	3921
Small-time	Taiwan	2700	4358	6564	3529	2971	16519	9984	2904	9238	8438

Table S13: Effective sample sizes under the DPG2 model. See Table S10 caption for notation.

Alignment	Island	Chain length (10^6)	posterior	likelihood	prior	μ	Height	κ	N_e	m	π
Large-active	New Zealand	328	170	263	294	293	827	3888	102	2583	2229
Large-active	Australia	357	396	586	412	489	2905	5104	189	1499	1730
Large-active	Iceland	257	100	180	**87**	162	1675	3669	**57**	1246	1001
Large-active	Taiwan	439	359	985	328	286	739	6557	161	749	538
Large-time	New Zealand	315	386	**26**	222	351	2372	5714	**78**	2427	2408
Large-time	Australia	320	580	1139	428	806	2647	5170	460	904	848
Large-time	Iceland	241	645	791	561	967	3426	4569	254	1326	751
Large-time	Taiwan	418	670	477	644	530	1038	6445	188	1736	1694
Small-active	New Zealand	180	626	406	474	556	1019	2727	651	214	146
Small-active	Australia	180	662	1000	659	642	1408	2774	431	1593	1456
Small-active	Iceland	180	921	1738	800	566	2135	1840	256	676	615
Small-active	Taiwan	900	5977	9534	4677	4292	5677	16069	2076	5533	5474
Small-time	New Zealand	180	1201	**81**	1037	551	1547	3061	370	1018	990
Small-time	Australia	180	797	662	642	768	2886	2892	445	687	748
Small-time	Iceland	180	807	217	500	521	3600	2835	967	2134	1948
Small-time	Taiwan	817	3503	4816	2634	2742	12256	12609	3213	2686	2415

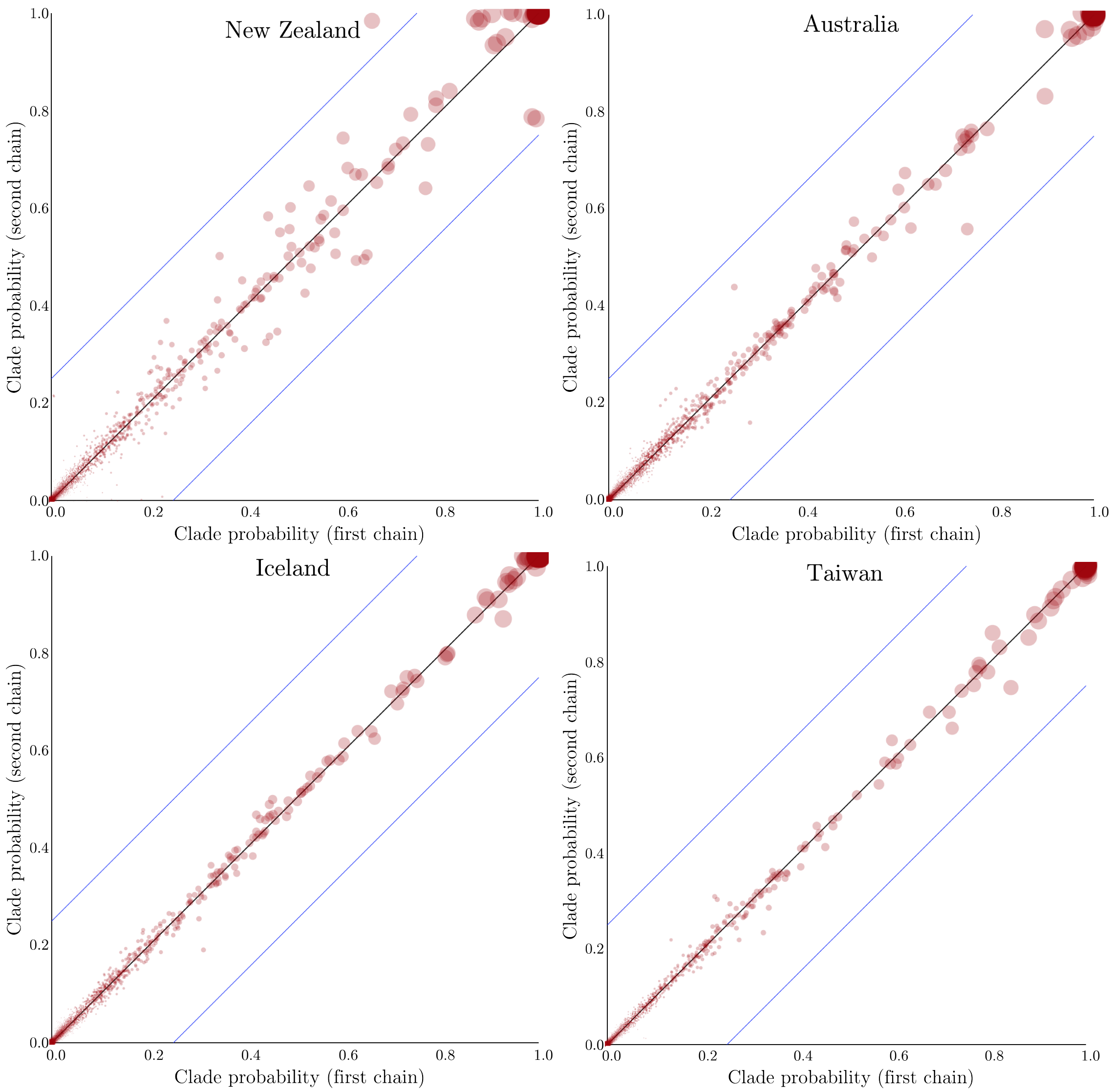

Fig. S4: The points on each plot are clade posterior probabilities from two independent MTBD analyses (using the "small-time" alignments). The size of each point is proportional to clade probability. These analysis indicates that tree topologies from independent MCMC chains converged to similar posterior distributions, with most probability differences being significantly less than than 0.25 (blue lines).

6 Supplementary results

6.1 Parameter estimates

Changes in posterior estimates for MTBD parameters over time intervals (for the "small-active" alignment) are presented in Fig. S5. These results indicate that the rate of becoming non-infectious $b = \mu + \psi$ increases over time in all four islands. This results from two mechanisms: 1) in increase in sampling rate ψ, likely due to higher rates of SARS-CoV-2 genomic sequencing (Fig. S8), and 2) an increase in death rate μ, likely due to improved measures of self-isolation and/or quarantine enforced by the respective governments (Fig. S9).

Clock rate and root height estimates vary slightly among differing models and subsampling schemes (Fig. S10). However, the four sampling methods yielded similar results for key MTBD parameters, suggesting that the MTBD analysis was not sensitive to subsampling methods (Fig. S11, S12, S13, and S14).

6.2 Introductions through time

The first step in quantifying SARS-CoV-2 introductions into the four $\mathcal{IS}$ demes is to carry out ancestral state reconstruction (ASR). The main goal of ASR is to sample ancestral states at internal nodes so that states from adjacent nodes can be compared: if the state of a parent node is $\mathcal{RW}$ and that of its child is $\mathcal{IS}$, an introduction is inferred. (The sampling procedure is done either simultaneously with the sampling of the tree, or during post-processing by using the parameter values logged during MCMC.) All branches of the tree can then be parsed and introductions annotated according to their chronological distribution, which allows one to plot the number of introductions over time (Fig. S15).

Under all models, ASR is carried out by traversing the tree backward in time, followed by a forward pass during which ancestral states are sampled (note that in a Bayesian framework we sample states at internal nodes, instead of trying to find the marginal or joint collection of states that maximize the likelihood, e.g., Pupko et al., 2000; Yang, 2014). In the case of DPG, DPG2, and MTBD, the tree is peeled backward so that partial likelihoods can be obtained at internal nodes and at the root, and then stochastic mapping is carried out forward in time (Nielsen, 2002; Freyman and Höhna, 2019). Under the SC model, ASR follows an approach similar in spirit to that of Pearl (1982); the procedure has been previously, and thoroughly detailed elsewhere (Müller et al., 2018).

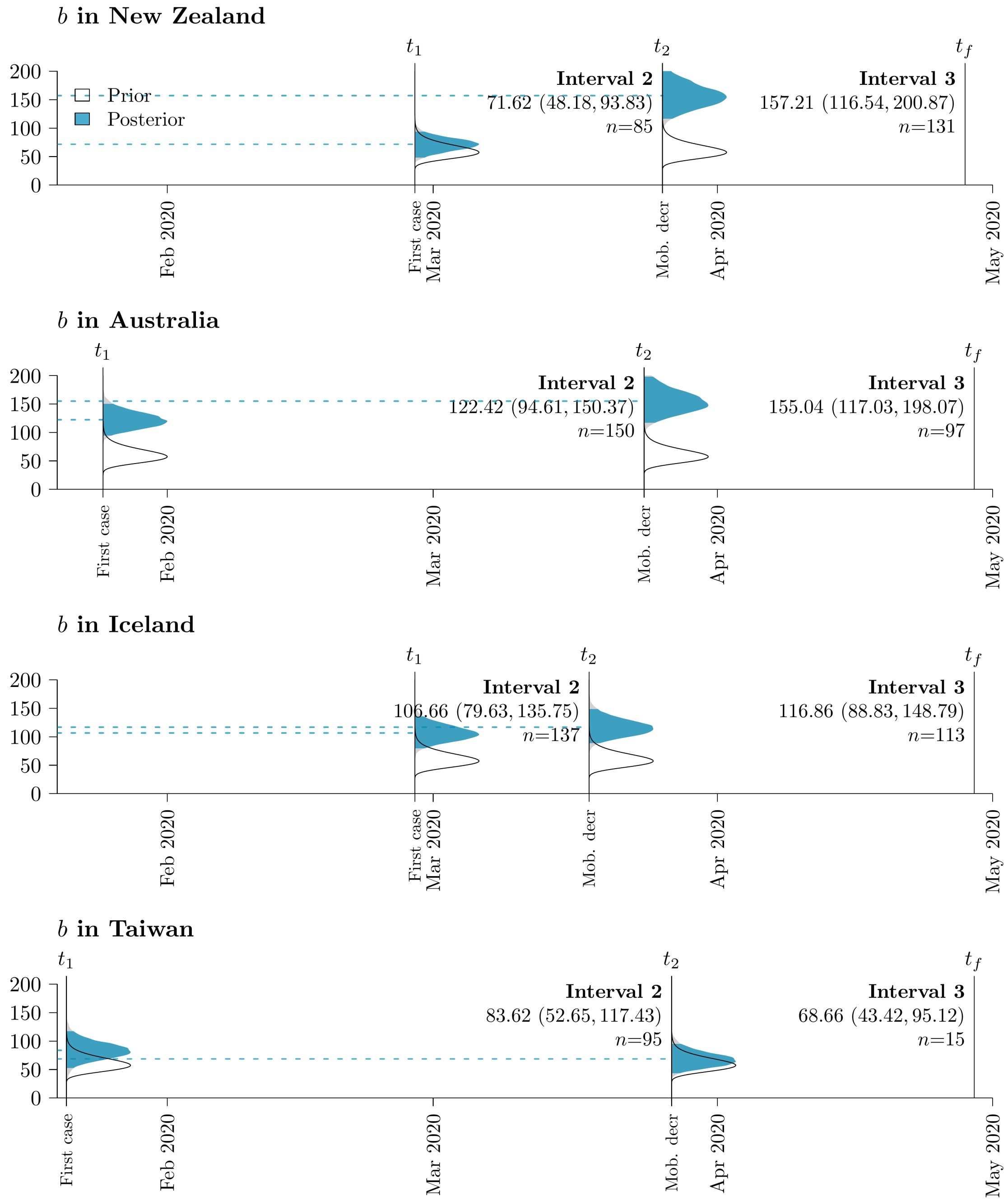

Fig. S5: Posterior distribution of the rate of becoming non-infectious b across the two epochs following the first reported case in the respective island. Relative prior densities and 95 % HPD intervals (blue) are displayed along the x-axis, with the mean posterior estimate indicated with a dashed line. The number of samples n from the specified country within the epoch is reported.

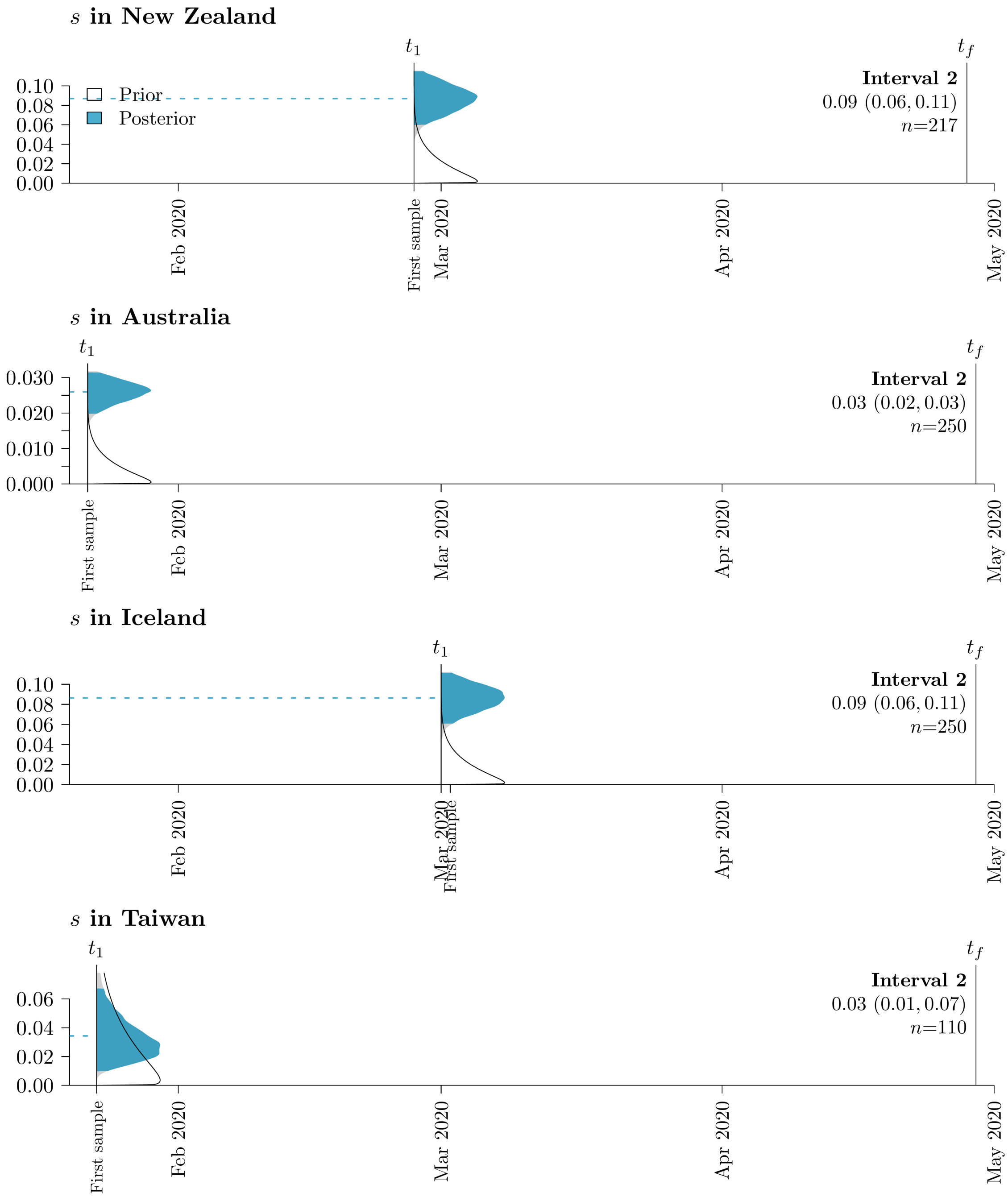

Fig. S6: Posterior distribution of sampling proportions s after the epoch following the first sample. s is held constant at 0 throughout the interval before the first sample. See Fig. S5 for further details on figure notation.

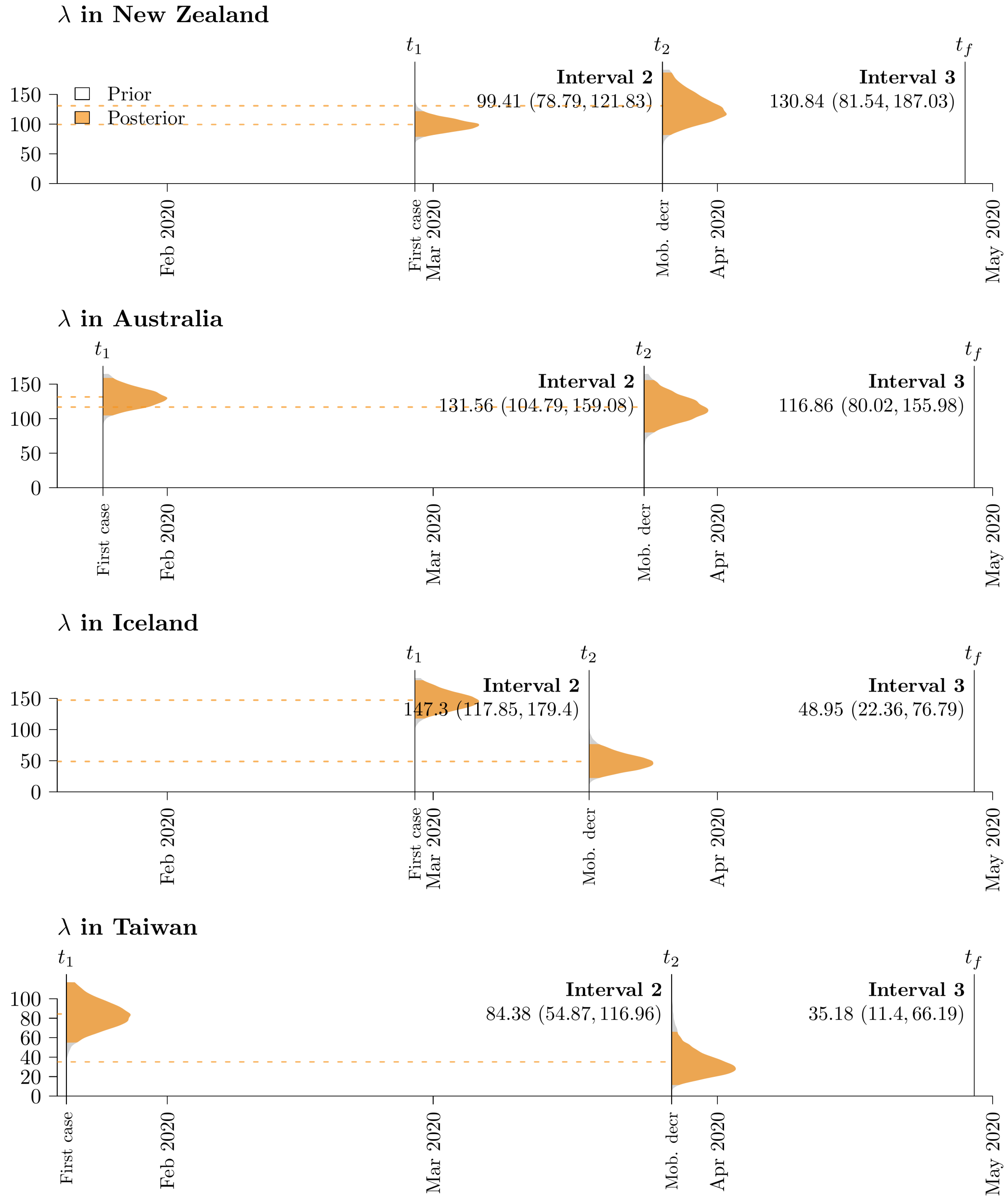

Fig. S7: Posterior distribution of the birth rate λ, following the first reported case. Although λ is not directly estimated as a model parameter, it can be calculated using $\lambda = \frac{R_e}{b}$. See Fig. S5 for further details on figure notation.

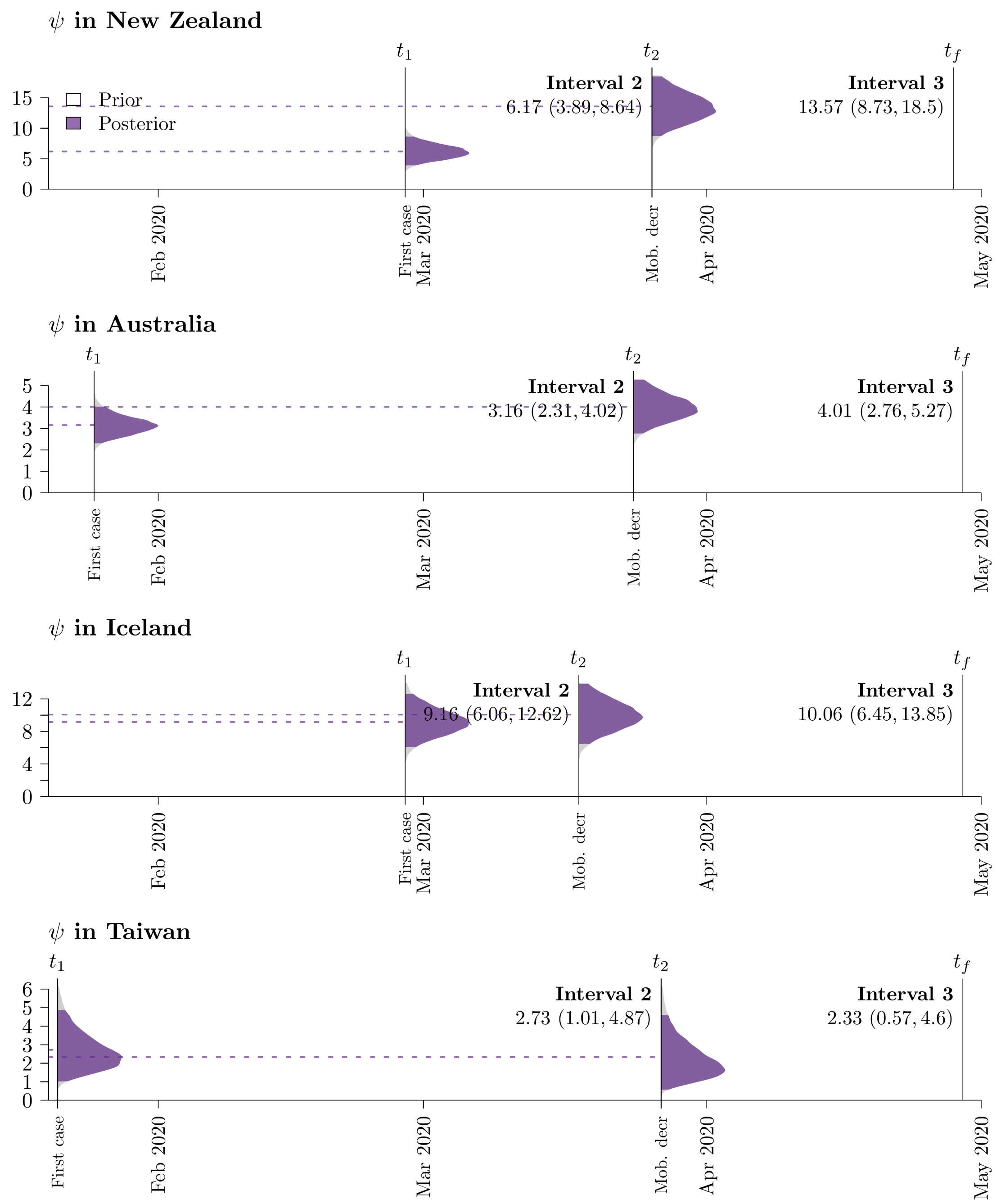

Fig. S8: Posterior distribution of the sampling rate ψ, following the first reported case. Although ψ is not directly estimated as a model parameter, it can be calculated using $\psi = sb$ (when $r = 1$). See Fig. S5 for further details on figure notation.

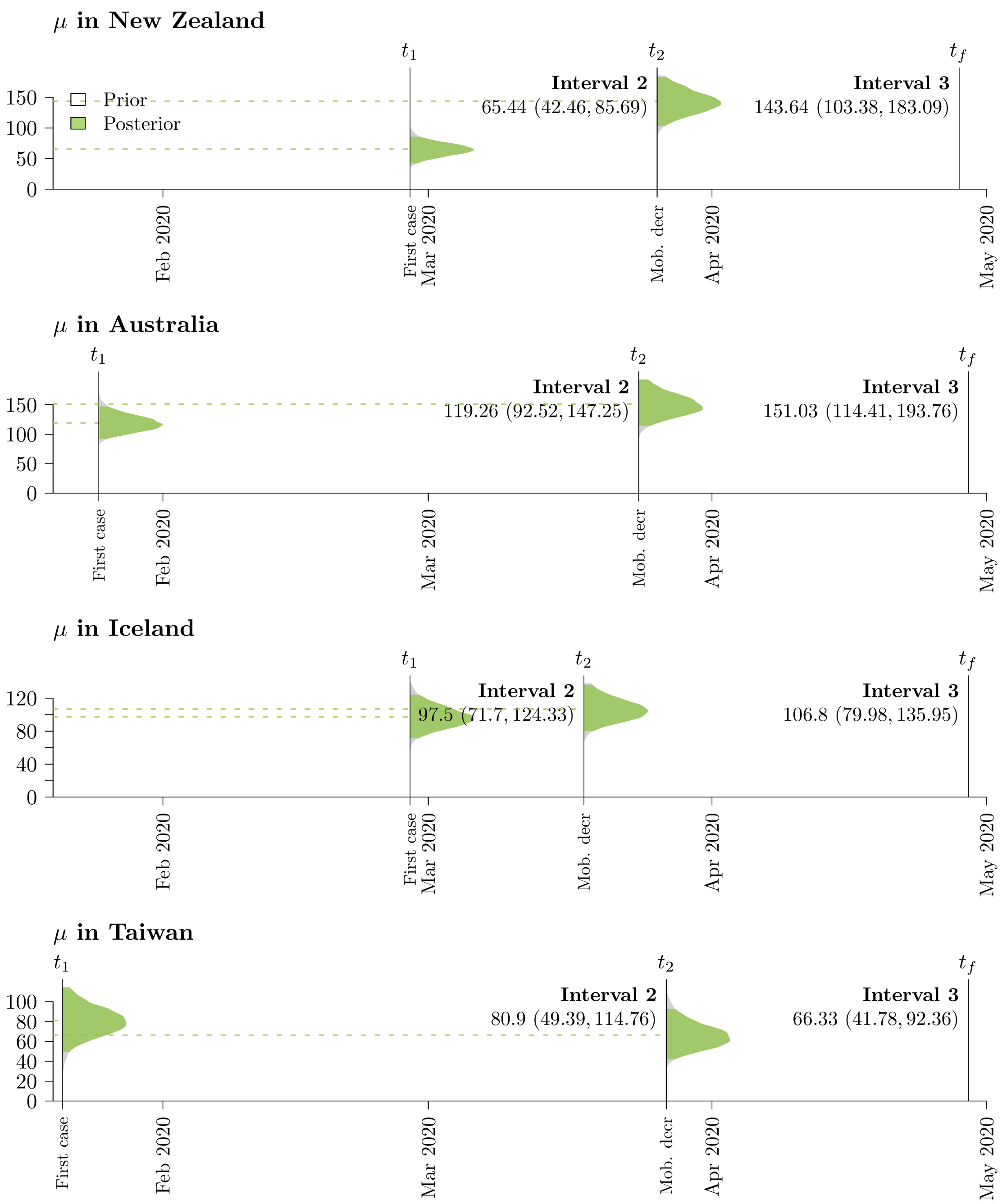

Fig. S9: Posterior distribution of the death rate μ, following the first reported case. Although μ is not directly estimated as a model parameter, it can be calculated using $\lambda = b - sb$ (when $r = 1$). See Fig. S5 for further details on figure notation.

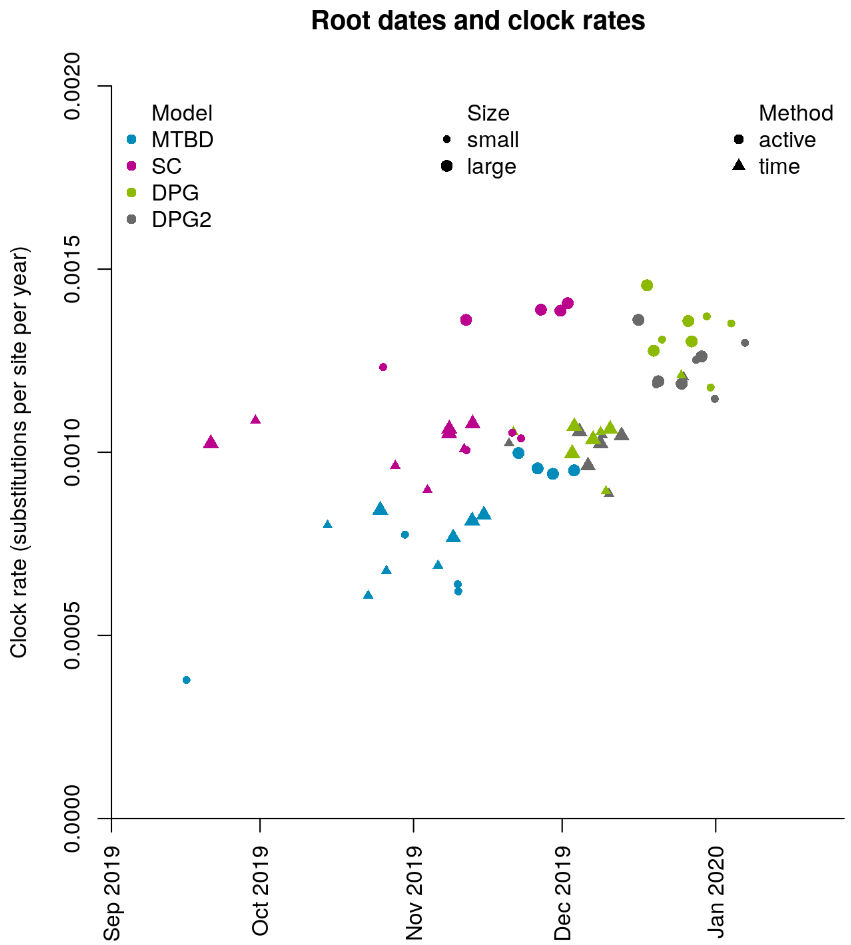

Fig. S10: Comparison of mean root height and clock rate estimates across the 64 combinations of sampling methods, models, and islands. These results show that MTBD and SC are less robust to changes in the sample and tend to give, on average, older tree heights. In contrast, DPG and DPG2 both give very late estimates for the root (late Dec - early Jan) when the active sampling method is employed, thus providing further evidence that the active method is not suitable for these models. For MTBD, the "small" datasets yield lower clock rates estimates.

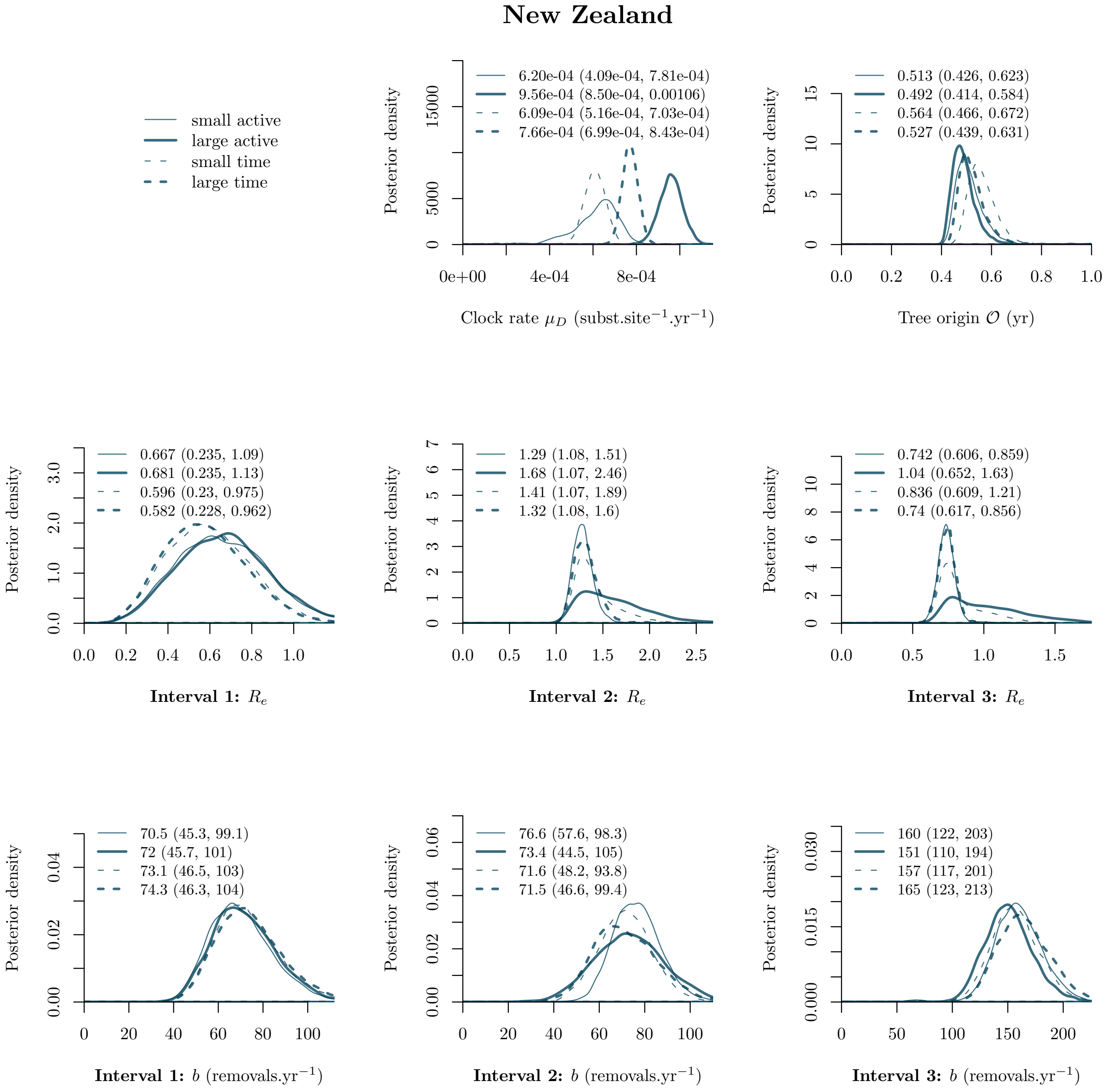

Fig. S11: Comparison of the four subsampling methods for New Zealand alignments. Posterior distributions of key parameters from the MTBD analyses are presented above. Alignment-specific mean estimates and 95% highest posterior density intervals are printed above the plots.

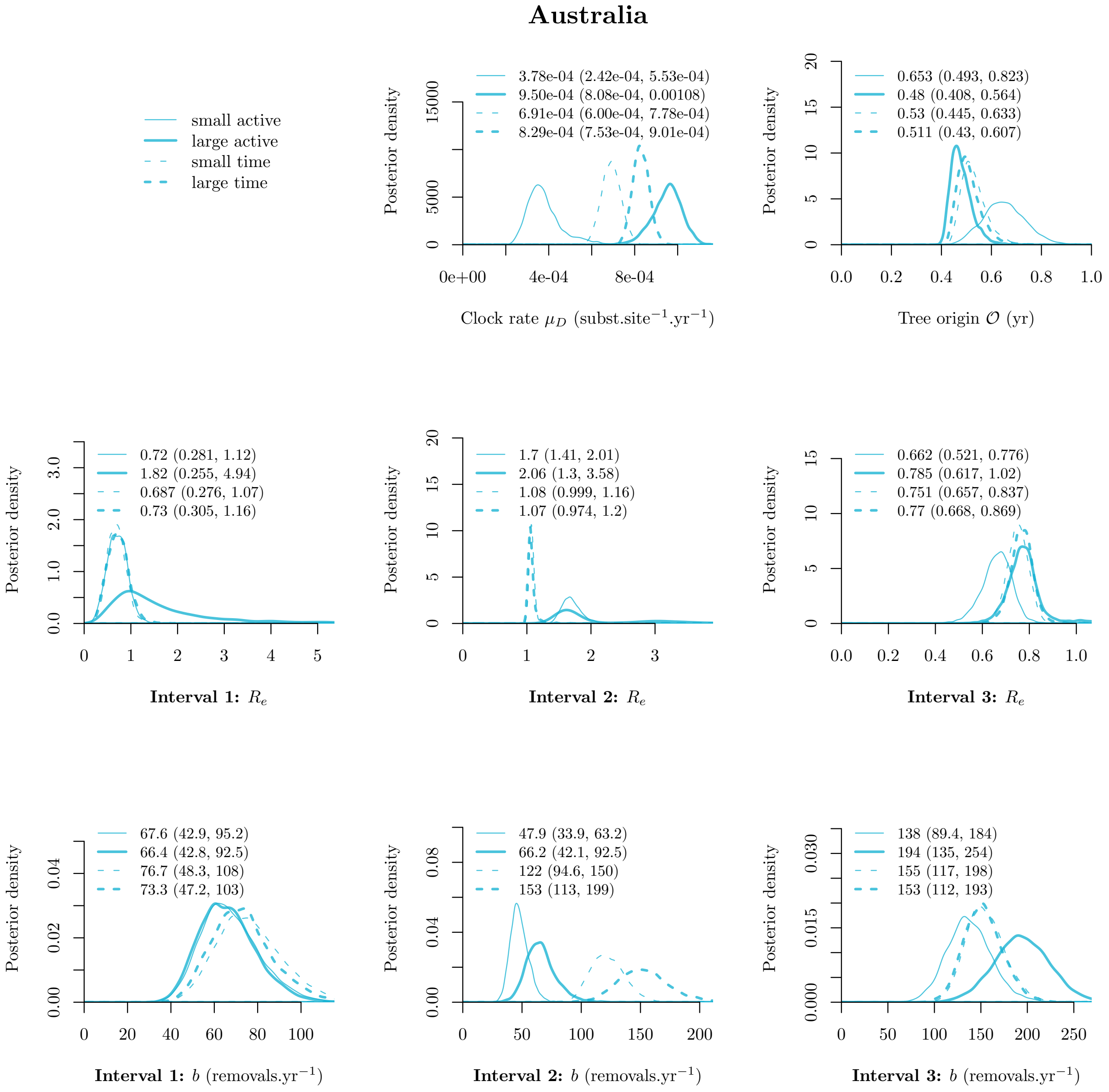

Fig. S12: Comparison of subsampling methods for Australia alignments. See Fig. S11 for further details.

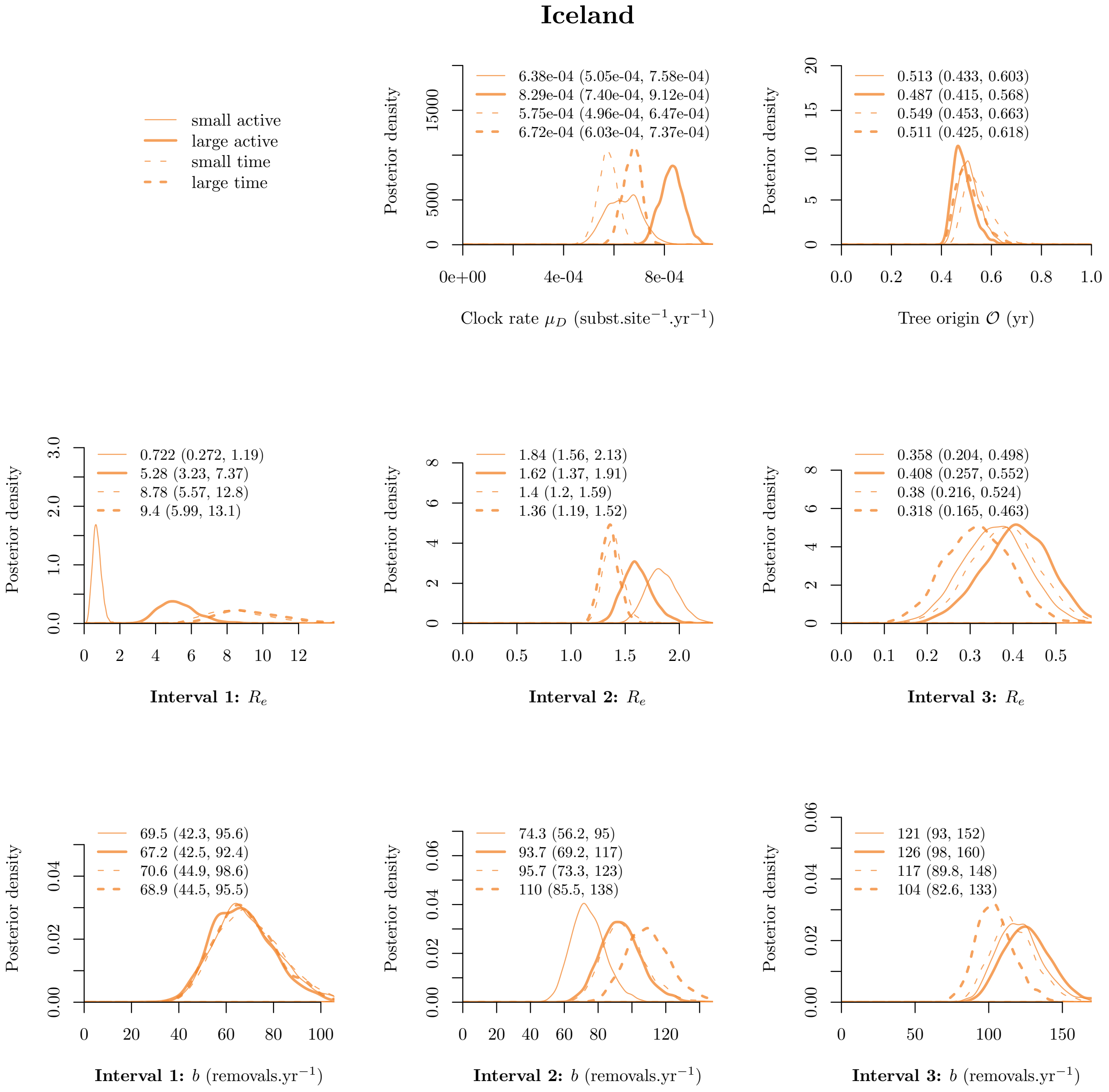

Fig. S13: Comparison of subsampling methods for Iceland alignments. See Fig. S11 for further details.

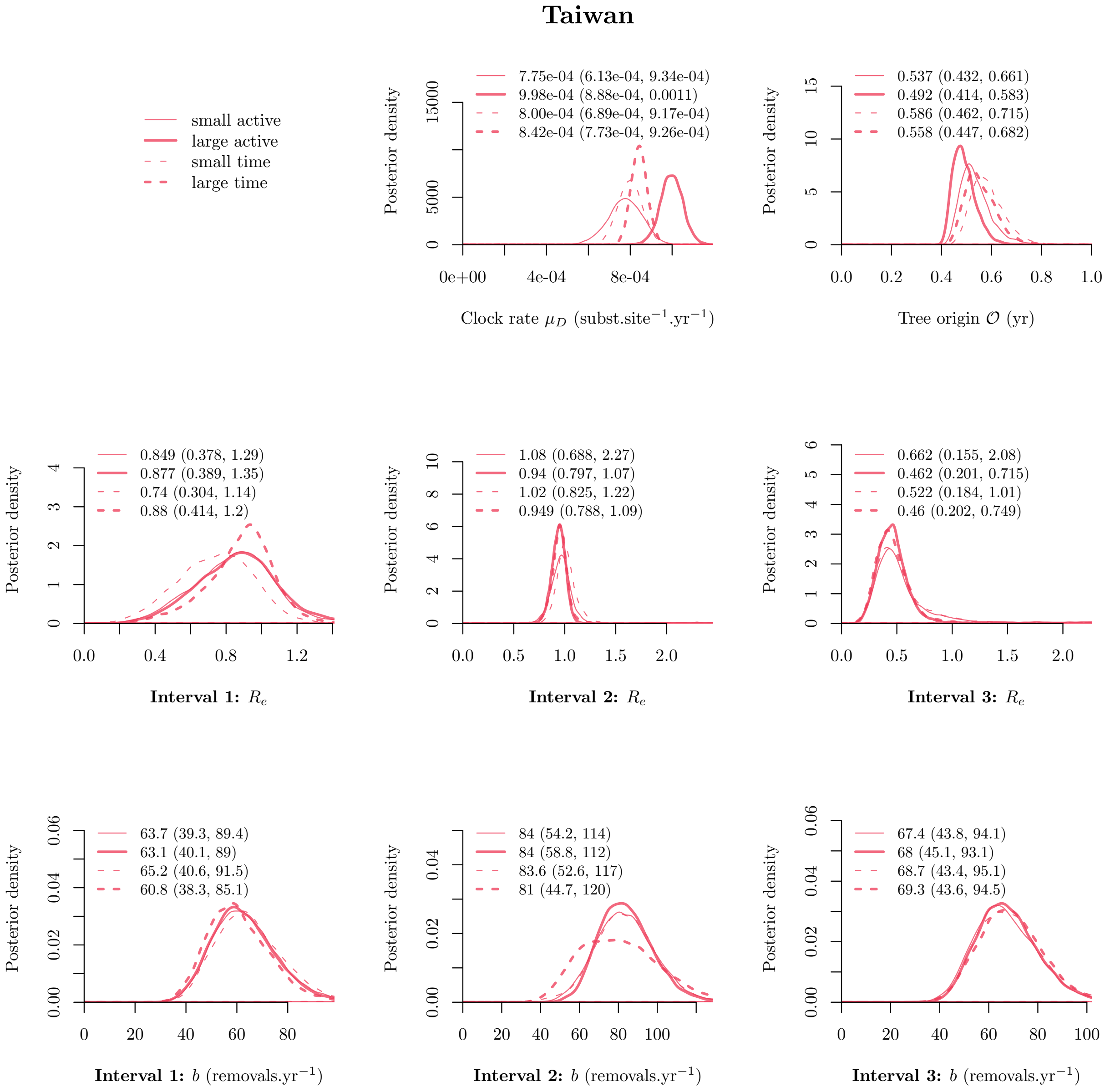

Fig. S14: Comparison of subsampling methods for Taiwan alignments. See Fig. S11 for further details.

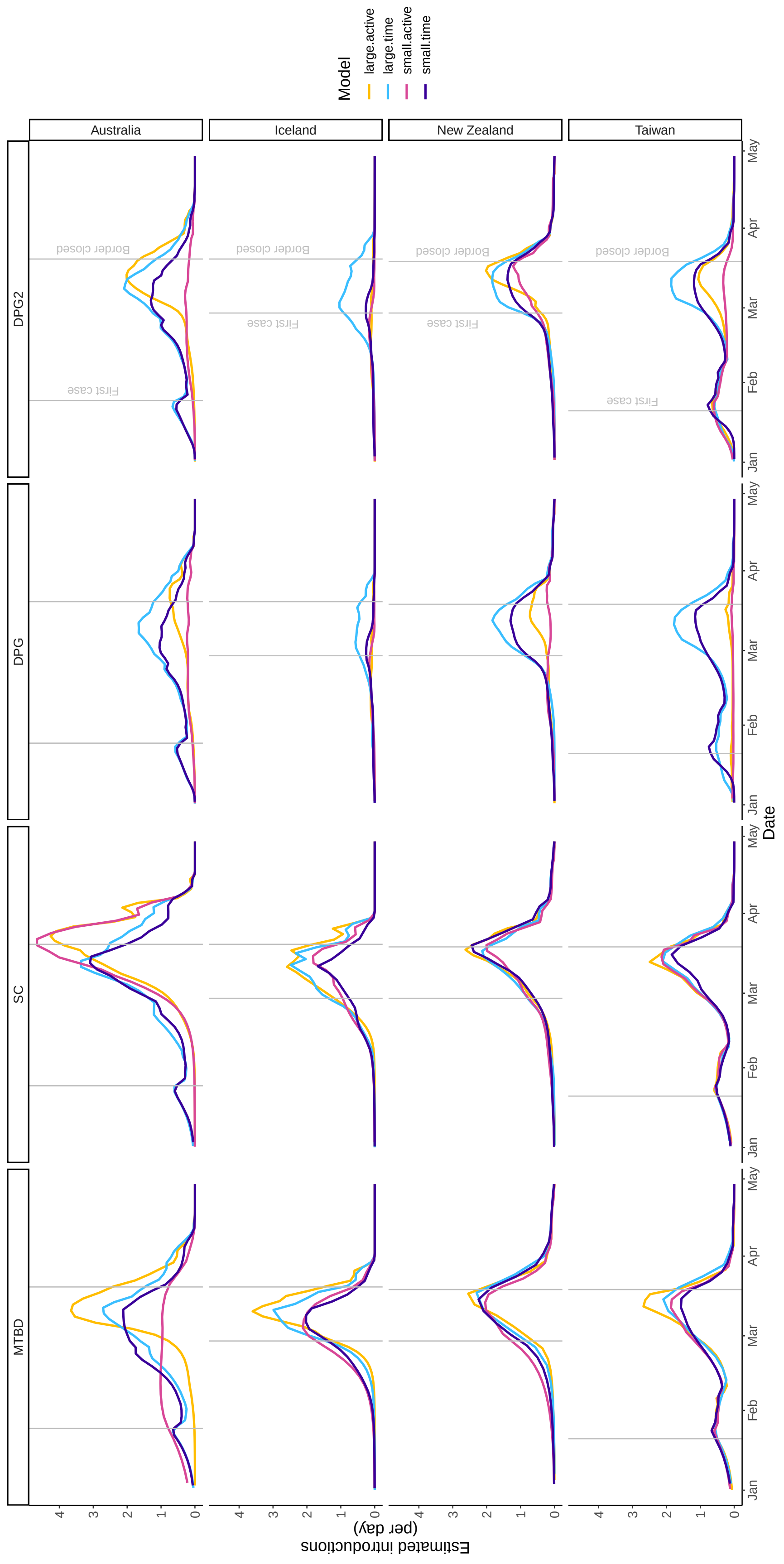

Fig. S15: Comparison of subsampling methods on SARS-CoV-2 introductions over time.

Table S14: Estimated number of imports and exports (mean and corresponding and 95% HPD intervals) into each target island deme $\mathcal{IS}$ (using the "small-time" subsampling protocol). *Overseas (total)* assumes that the sample is representative of the total proportion of cases linked to overseas travel in Table 1 of the main article. *Overseas (sample)* counts the number of samples that have been marked as having recent overseas travel in the GISAID sequence metadata Shu and McCauley (2017). These counts are likely influenced by missing data. Estimates are highlighted in bold if the expected number is within the 95% HPD interval of either of these two methods.

Island $\mathcal{IS}$	Model	Estimated imports	Estimated exports
	Overseas (total)	84	
	Overseas (sample)	49	
New Zealand	DPG	**41 [29,51]**	$14[5,23]$
	DPG2	**41 [30,50]**	$11[4,20]$
	SC	**58 [48,67]**	$0[0,0]$
	MTBD	$63[54,72]$	$1.4[0,4]$
	Overseas (total)	159	
	Overseas (sample)		
Australia	DPG	$49[33,63]$	$34[17,50]$
	DPG2	$52[38,65]$	$31[16,44]$
	SC	$98[83,111]$	$0[0,0]$
	MTBD	$87[72,100]$	$0.68[0,3]$
	Overseas (total)	47	
	Overseas (sample)	68	
Iceland	DPG	$6.9[6,9]$	$36[26,46]$
	DPG2	$7[5,9]$	$43[32,55]$
	SC	**37 [25,48]**	$0[0,0]$
	MTBD	**49 [31,64]**	$0.95[0,3]$
	Overseas (total)	99	
	Overseas (sample)	25	
Taiwan	DPG	$48[37,58]$	$21[6,36]$
	DPG2	$49[38,58]$	$19[6,32]$
	SC	$57[47,68]$	$0[0,0]$
	MTBD	$65[53,76]$	$0.98[0,4]$

Table S15: Estimated imports and exports using the "large-time" method. See Table S14 caption for details.

Island $\mathcal{IS}$	Model	Estimated imports	Estimated exports
	Overseas (total)	84	
	Overseas (sample)	49	
New Zealand	DPG	**47 [39,55]**	$1.3[0,4]$
	DPG2	**48 [39,55]**	$2.3[0,5]$
	SC	**55 [47,64]**	$0[0,0]$
	MTBD	$60[50,67]$	$0.52[0,1]$
	Overseas (total)	159	
	Overseas (sample)		
Australia	DPG	$68[55,80]$	$47[27,67]$
	DPG2	$70[56,80]$	$47[29,64]$
	SC	$110[100,126]$	$0[0,0]$
	MTBD	$92[77,104]$	$2.6[0,9]$
	Overseas (total)	47	
	Overseas (sample)	68	
Iceland	DPG	$19[9,27]$	$65[40,87]$
	DPG2	$28[18,37]$	$54[31,76]$
	SC	**60 [49,68]**	$0[0,0]$
	MTBD	**62 [53,71]**	$0.7[0,3]$
	Overseas (total)	99	
	Overseas (sample)	25	
Taiwan	DPG	$59[49,66]$	$23[6,40]$
	DPG2	$60[50,67]$	$29[15,44]$
	SC	$67[59,74]$	$0[0,0]$
	MTBD	$69[60,77]$	$0.93[0,3]$

Table S16: Estimated imports and exports using the "small-active" method. See Table S14 caption for details.

Island $\mathcal{IS}$	Model	Estimated imports	Estimated exports
	Overseas (total)	84	
	Overseas (sample)	49	
New Zealand	DPG	$13[7, 18]$	$41[29, 52]$
	DPG2	**32 [9,49]**	$20[1, 44]$
	SC	**54 [43,62]**	$0[0, 0]$
	MTBD	$64[55, 71]$	$0.13[0, 1]$
	Overseas (total)	159	
	Overseas (sample)		
Australia	DPG	$14[8, 20]$	$68[52, 80]$
	DPG2	$14[9, 19]$	$82[66, 96]$
	SC	$120[99, 135]$	$0[0, 0]$
	MTBD	$72[51, 91]$	$5.6[0, 17]$
	Overseas (total)	47	
	Overseas (sample)	68	
Iceland	DPG	$4.4[4, 6]$	$29[21, 36]$
	DPG2	$4.6[4, 6]$	$68[49, 84]$
	SC	**47 [36,60]**	$0[0, 0]$
	MTBD	**57 [43,69]**	$0.28[0, 1]$
	Overseas (total)	99	
	Overseas (sample)	25	
Taiwan	DPG	$4.3[1, 10]$	$80[63, 98]$
	DPG2	**26 [16,35]**	$90[68, 110]$
	SC	$66[55, 77]$	$0[0, 0]$
	MTBD	**69 [24,82]**	$11[0, 116]$

Table S17: Estimated imports and exports using the "large-active" method. See Table S14 caption for details.

Island $\mathcal{IS}$	Model	Estimated imports	Estimated exports
	Overseas (total)	84	
	Overseas (sample)	49	
New Zealand	DPG	$22[11,36]$	$48[13,77]$
	DPG2	$40[31,48]$	$17[7,26]$
	SC	**58 [49,67]**	$0[0,0]$
	MTBD	**54 [42,65]**	$9.7[0,20]$
	Overseas (total)	159	
	Overseas (sample)		
Australia	DPG	$27[13,43]$	$67[28,101]$
	DPG2	$50[35,65]$	$38[19,57]$
	SC	$110[88,120]$	$0.0012[0,0]$
	MTBD	$82[51,112]$	$22[0,75]$
	Overseas (total)	47	
	Overseas (sample)	68	
Iceland	DPG	$4.9[4,7]$	$55[41,69]$
	DPG2	$5.1[4,7]$	$100[66,132]$
	SC	**62 [48,74]**	$0[0,0]$
	MTBD	**65 [53,77]**	$1[0,3]$
	Overseas (total)	99	
	Overseas (sample)	25	
Taiwan	DPG	$8.2[1,16]$	$96[63,126]$
	DPG2	**40 [23,55]**	$120[60,181]$
	SC	$68[59,77]$	$0[0,0]$
	MTBD	$72[63,80]$	$0.8[0,3]$

References

Apple. 2020. Mobility trends reports. [Online on https://www.apple.com/covid19/mobility; posted on 25 May 2020].

Bielejec, F., P. Lemey, G. Baele, A. Rambaut, and M. A. Suchard. 2014. Inferring heterogeneous evolutionary processes through time: from sequence substitution to phylogeography. Syst. Biol. 63:493–504.

Bolger, A. M., M. Lohse, and B. Usadel. 2014. Trimmomatic: a flexible trimmer for illumina sequence data. Bioinformatics 30:2114–2120.

Bouckaert, R., T. G. Vaughan, J. Barido-Sottani, S. Duchêne, M. Fourment, A. Gavryushkina, J. Heled, G. Jones, D. Kühnert, N. De Maio, et al. 2019. BEAST 2.5: An advanced software platform for bayesian evolutionary analysis. PLoS Comput. Biol. 15:e1006650.

Bouckaert, R. R. and A. J. Drummond. 2017. bModelTest: Bayesian phylogenetic site model averaging and model comparison. BMC Evol. Biol. 17:42.

Day, M. 2020a. COVID-19: four fifths of cases are asymptomatic, China figures indicate. BMJ 369:m1375.

Day, M. 2020b. COVID-19: identifying and isolating asymptomatic people helped eliminate virus in Italian village. BMJ 368:m1165.

Drummond, A. J., A. Rambaut, B. Shapiro, and O. G. Pybus. 2005. Bayesian coalescent inference of past population dynamics from molecular sequences. Mol. Biol. Evol. 22:1185–1192.

Eden, J.-S., R. Rockett, I. Carter, H. Rahman, J. De Ligt, J. Hadfield, M. Storey, X. Ren, R. Tulloch, K. Basile, et al. 2020. An emergent clade of SARS-CoV-2 linked to returned travellers from Iran. Virus Evol. 6:veaa027.

Freyman, W. A. and S. Höhna. 2019. Stochastic character mapping of state-dependent diversification reveals the tempo of evolutionary decline in self-compatible Onagraceae lineages. Syst. Biol. 68:505–519.

Garrison, E. 2014. A C++ library for parsing and manipulating vcf files. [Online on https://github.com/vcflib/vcflib].

Gelman, A. et al. 2006. Prior distributions for variance parameters in hierarchical models (comment on article by Browne and Draper). Bayesian Anal. 1:515–534.

Grubaugh, N. D., K. Gangavarapu, J. Quick, N. L. Matteson, J. G. De Jesus, B. J. Main, A. L. Tan, L. M. Paul, D. E. Brackney, S. Grewal, et al. 2019. An amplicon-based sequencing framework for accurately measuring intrahost virus diversity using primalseq and ivar. Genome Biol. 20:1–19.

Hadfield, J., C. Megill, S. M. Bell, J. Huddleston, B. Potter, C. Callender, P. Sagulenko, T. Bedford, and R. A. Neher. 2018. Nextstrain: real-time tracking of pathogen evolution. Bioinformatics 34:4121–4123.

Hale, T., S. Webster, A. Petherick, T. Phillips, and B. Kira. 2020. Oxford COVID-19 government response tracker. Blavatnik School of Government. [Online on `https://github.com/OxCGRT/covid-policy-tracker`; posted on 4 June 2020].

Hoffman, M. D. and A. Gelman. 2014. The No-U-Turn sampler: adaptively setting path lengths in Hamiltonian Monte Carlo. J. Mach. Learn. Res. 15:1593–1623.

Katoh, K., G. Asimenos, and H. Toh. 2009. Multiple alignment of DNA sequences with MAFFT. Pages 39–64 *in* Bioinformatics for DNA sequence analysis. Springer.

Kühnert, D., T. Stadler, T. G. Vaughan, and A. J. Drummond. 2016. Phylodynamics with migration: a computational framework to quantify population structure from genomic data. Mol. Biol. Evol. 33:2102–2116.

Lai, A., A. Bergna, C. Acciarri, M. Galli, and G. Zehender. 2020. Early phylogenetic estimate of the effective reproduction number of SARS-CoV-2. J. Med. Virol. 92:675–679.

Lemey, P., A. Rambaut, A. J. Drummond, and M. A. Suchard. 2009. Bayesian phylogeography finds its roots. PLoS Comp. Biol. 5.

Li, R., S. Pei, B. Chen, Y. Song, T. Zhang, W. Yang, and J. Shaman. 2020a. Substantial undocumented infection facilitates the rapid dissemination of novel coronavirus (SARS-CoV-2). Science 368:489–493.

Li, X., J. Zai, Q. Zhao, Q. Nie, Y. Li, B. T. Foley, and A. Chaillon. 2020b. Evolutionary history, potential intermediate animal host, and cross-species analyses of SARS-CoV-2. J. Med. Virol. 92:602–611.

Loman, N., W. Rowe, and A. Rambaut. 2020. nCoV-2019 novel coronavirus bioinformatics protocol. [Online on `https://artic.network/ncov-2019/ncov2019-bioinformatics-sop.html`; posted on 23 January 2020].

Lu, F. S., A. Nguyen, N. Link, and M. Santillana. 2020. Estimating the prevalence of COVID-19 in the United States: three complementary approaches. medRxiv .

Müller, N. F. and R. Bouckaert. 2019. Coupled MCMC in BEAST 2. bioRxiv .

Müller, N. F., D. Rasmussen, and T. Stadler. 2018. MASCOT: parameter and state inference under the marginal structured coalescent approximation. Bioinformatics 34:3843–3848.

Nielsen, R. 2002. Mapping mutations on phylogenies. Syst. Biol. 51:729–39.

Pearl, J. 1982. Reverend Bayes on inference engines: a distributed hierarchical approach. University of California, Los Angeles.

Pupko, T., R. Shamir, and D. Graur. 2000. A fast algorithm for joint reconstruction of ancestral amino acid sequences. Mol. Biol. Evol. 17:890–896.

Rambaut, A. 2020. Phylodynamic analysis | 176 genomes | 6 mar 2020. [Online on `http://virological.org/t/phylodynamic-analysis-176-genomes-6-mar-2020/356`; posted on 6 March 2020].

Sagulenko, P., V. Puller, and R. A. Neher. 2018. TreeTime: Maximum-likelihood phylodynamic analysis. Virus Evol. 4.

Salvatier, J., T. V. Wiecki, and C. Fonnesbeck. 2016. Probabilistic programming in Python using PyMC3. PeerJ Comput. Sci. 2:e55.

Shu, Y. and J. McCauley. 2017. GISAID: Global initiative on sharing all influenza data – from vision to reality. Euro. Surveill. 22:30494.

Yang, Z. 2014. Molecular evolution: a statistical approach. Oxford University Press.
